# Statins significantly reduce mortality in patients receiving clopidogrel without affecting platelet activation and aggregation: a systematic review and meta-analysis

**DOI:** 10.1186/s12944-019-1053-0

**Published:** 2019-05-24

**Authors:** Ke An, Rong Huang, Sai Tian, Dan Guo, Jiaqi Wang, Hongyan Lin, Shaohua Wang

**Affiliations:** 1 Department of Endocrinology, Affiliated Zhongda Hospital of Southeast University, No. 87 DingJiaQiao Road, Nanjing, 210009 People’s Republic of China; 2 Medical School of Southeast University, Nanjing, 210009 People’s Republic of China

**Keywords:** Clopidogrel, Statins, Meta-analysis, Mortality, Platelet aggregation, Lipid

## Abstract

**Background:**

Combination of statins and clopidogrel is frequently administered in patients with coronary artery disease (CAD). They are mainly activated and eliminated in the liver by cytochrome P450 isoenzyme 3A4 (CYP3A4). The aim was to clarify whether the coadministration of clopidogrel and statins attenuate respective efficacy.

**Methods:**

PubMed, Embase, the Cochrane Library, Web of Science and Clinical Trials. gov were searched for until August 2018. Randomized controlled trials (RCTs) and cohort studies were taken into quality evaluation. Data were pooled using random effect models to estimate standard mean difference (SMD) or risk ratio (RR) with 95% confidence interval (CI).

**Results:**

In total, 28 studies representing 25,267 participants were included. Statins reduce the mortality of patients administered clopidogrel (RR 0.54; 95% CI 0.40,0.74; *p* = 0.000), no differences were found in platelet aggregation (PA) (SMD 0.02; 95% CI -0.38,0.42; *p* = 0.920) and the expressions of P-selectin (SMD -0.04; 95% CI -0.14,0.05; *p* = 0.346), CD40L (SMD 0.09; 95% CI -0.29,0.48; *p* = 0.633), CD63 (SMD 0.09; 95% CI -0.01,0.19; *p* = 0.079) and PAC-1 (SMD 0.03; 95% CI -0.08,0.13; *p* = 0.633). Furthermore, CYP3A4 metabolized or non-CYP3A4 metabolized statins have no discrepancies in PA (SMD 0.13; 95% CI -0.31,0.58; *p* = 0.556), P-selectin (SMD 0.17; 95% CI -0.16,0.51; *p* = 0310), death (RR 0.89; 95% CI 0.38,2.07; *p* = 0.791), except for triglyceride (TG) (SMD -0.19; 95% CI –0.33,-0.06; *p* = 0.005).

**Conclusions:**

This meta-analysis confirmed that statins reduce mortality in patients undergoing clopidogrel treatment without affecting platelet activation and aggregation.

**Electronic supplementary material:**

The online version of this article (10.1186/s12944-019-1053-0) contains supplementary material, which is available to authorized users.

## Introduction

Statins, beyond the low-density lipoprotein lowering and high-density lipoprotein raising, are widely used in the medical therapy of coronary artery disease (CAD) for the dramatic reduction of cardiovascular events [1, 2]. In addition, statins are predominantly metabolized by certain cytochrome P450 isoenzyme 3A4 (CYP3A4) (atorvastatin, lovastatin, simvastatin, cerivastatin), whereas others are not substrates of CYP3A4 (pravastatin, rosuvastatin) [3]. Clopidogrel, the most commonly used drug of CAD, is an inactive prodrug requiring oxidation by the hepatic cytochrome P450 isoenzyme system to exert an active metabolite, especially CYP3A4 system, which irreversibly blocks the platelet P2Y12 receptor [4].

Since the concomitant use of clopidogrel and statin is a key element in the therapy of CAD, and given that they share the same metabolic way, there is a growing interest in exploring the potential interaction between clopidogrel and statin on platelet activation, platelet aggregation (PA), lipid control, and clinical outcomes. However, available studies on this topic have shown inconsistent and inconclusive findings. Several studies have reported that statins significantly had reduced antiaggregatory effect on clopidogrel, especially CYP3A4 metabolized statins [5–7], whereas some other reports have indicated that therapy with statins did not jeopardize the antiplatelet activity of clopidogrel [8–12]. Furthermore, a number of trials have shown that statins did not have an impact on the clinical outcome of the clopidogrel treatment after percutaneous coronary intervention (PCI) [11, 13–16], while others have noted that patients taking atorvastatin and clopidogrel had the increased risk of major adverse events [17].

Drug interaction affecting either the efficacy or safety of clopidogrel therapy is of paramount importance. Therefore, we conducted this meta-analysis by systematically incorporating the latest evidence with a primary focus on the impacts of antiplatelet function of clopidogrel by the administration of statin in patients with CAD as well as on the investigation of lipid-lowering impact of statin when it was combined with clopidogrel. Moreover, since statins can be divided into CYP3A4 metabolized and non-CYP3A4 metabolized, our secondary aim was to assess whether clopidogrel recipients has distinct response to them. Finally, we address the question of whether statin regimens in combination of clopidogrel can have less clinical outcomes.

## Methods

### Data sources and search strategy

We searched PubMed, EMBASE, the Cochrane Library, Web of Science and Clinical Trials gov for reports published up to August 2018 using the search terms “(clopidogrel OR clopidogrel napadisilate OR platelet aggregation inhibitor OR purinergic P2Y receptor antagonist)” and “(statins OR hydroxymethylglutaryl-CoA reductase inhibitors OR HMG-CoA OR lipid lowering drugs OR atorvastatin OR fluvastatin OR lovastatin OR pravastatin OR rosuvastatin OR simvastatin)”. We restricted the search to “human species”. The searches were not restricted by the date of study publication, language of publication or age of study subjects. The details of search strategy of PubMed was provided in Additional file 1. Reports were further screened for inclusion by reviewing their titles, abstracts, or full texts. We also examined the reference lists of the identified articles previous meta-analyses to supplement the electronic search.

### Study selection

Two independent researchers accessed the articles based on the following inclusion criteria: (1) trials that reported the association between clopidogrel and statins and (2) participants were diagnosed with CAD. Any divergence was resolved by a reviewer.

### Data extraction and quality assessment

For included trials, the following data for each trial were extracted to identify whether the combination of clopidogrel and statin can affect respective efficacy: authors, year of publication, study properties (e.g. study design, sample size, population, intervention) and end points. We also recorded lipid metabolic variables (e.g. parameters of low-density lipoprotein cholesterol [LDL-C], high-density lipoprotein cholesterol [HDL-C], total cholesterol [TC], and triglyceride [TG]) to help understand the lipid-lowering process.

The quality of the cohort studies was evaluated using validated 9-star Newcastle-Ottawa Scale (NOS) [18], which assigns a maximum of 4 stars to the selection category, 2 stars for the comparability category, and 3 stars for the outcome category. Studies with NOS scores ≥7 were considered to be of high quality; otherwise, they were of low quality. The Cochrane Collaboration “Risk of Bias” tool [19], which includes items on selection bias, performance bias, detection bias, attrition bias, and reporting bias, was applied to evaluate the quality of RCTs. More details were presented in Tables 1 and 2. All the data collection and quality assessment were initially performed by one author, and another author checked the extracted data for accuracy.

**Table 1:** Characteristics of the 28 clinical trials

Study	Year	Study design	Sample size	Population	Intervention (mg/d)	Mean follow-up	End-points	NOS score
Lofti et al. [20]	2008	RCT	4162	ACS	G1: A80 + Clo G2: P40 + Clo	2 years	Death, MI, UA, stroke	–
Malmstrom et al. [21]	2009	RCT	69	CAD PCI	G1: R10–40 + Clo G2: A20–80 + Clo G3: S40 + Clo	16 weeks	PA, P-selectin, fibrinogen binding	–
Mitsios et al. [22]	2004	RCT	45	ACS	G1: A10 + Clo G2: P40 + Clo	5 weeks	PA, P-selectin, CD40L, lipids, ALT, AST, LDH, CK	–
Neubauer et al. [10]	2003	Prospective cohort	47	CAD	G1: S10/S20/A20/A40 + Clo G2: Clo	48 h	P-selectin	9
Ojeifo et al. [23]	2013	Cohort	4794	ACS	G1: Statin + Clo G2: Clo	450 days	Cardiovascular death, MI, stroke	7
Park et al. [24]	2016	RCT	3755	PCI	G1: CYP3A4 + Clo G2: non-CYP3A4 + Clo	1 month	PRU, death, MI, revascularization, stent thrombosis	–
Poyet et al. [25]	2010	RCT	138	ACS PCI	G1: A80 + Clo G2: R20 + Clo	1 month	PA, LDL-C, HDL-C, CRP	–
Schmidt et al. [26]	2012	Cohort	13,001	PCI	G1: Statin + Clo G2: Clo	12 months	MACE	7
Serebruany et al. [6]	2005	Prospective cohort	75	PCI	G1: A10–40 + Clo G2: other statins + Clo G3: Clo	24 h	PA, P-selectin, CD40L, CD63, PAC-1	8
Suh et al. [27]	2014	Prospective cohort	556	PCI	G1:A20 + Clo G2:R10 + Clo	6 months	PRU, lipids, MACE, death, MI, stent thrombosis, ischemic strike, target lesion revascularization	7
Toso et al. [28]	2017	Cohort	1053	ACS PCI	G1: Statin + Clo G2: Clo	1 year	PRI, MACE, death, Cardiac death, MI, stent thrombosis, stroke	7
Vinholt et al. [29]	2005	Cohort	66	CAD	G1: CYP3A4 + Clo G2: non-CYP3A4 + Clo	21 days	PA, TC, LDL-C	9
Wenaweser et al. [30]	2010	RCT	101	CAD PCI	G1: A40 + Clo G2: F80 + Clo	1 month	PA	–
Wenaweser et al. [31]	2007	RCT	73	PCI	G1: A20/P40 + Clo G2:Clo	1 month	PA, lipids, CRP	–
Zhang et al. [32]	2015	RCT	104	NSTE-ACS PCI	G1: A20 + Clo G2: Clo	6 months	IPA, P-selectin, TBX_2_, sCD40L	–
Gorchakova et al. [19]	2004	Cohort	180	CAD PCI	G1: A/S + Clo G2: Clo	At least 4 weeks	Maximal PA, Residual PA, P-selectin, CD61	8
Lim et al. [15]	2005	Prospective cohort	15,693	ACS	G1: statin + Clo G2: Clo	6 months	Rehospitalization, stroke, revascularization, death	9
Nagavi et al. [33]	2016	Prospective cohort	61	PCI	G1: A40 + Clo G2: R40 + Clo G3: Clo	24 h	PA	7
Riondino et al. [34]	2009	Cohort	105	PCI	G1: A20 + Clo G2: R10 + Clo G3: Clo	3 months	PA	8
Trenk et al. [11]	2008	Cohort	1395	PCI	G1: A + Clo G2: S + Clo G3: F + Clo G4: P + Clo G5: Clo	1 year	RPA, P-selectin, CD63, PAC-1, MACE, death, MI, target lesion reintervention	7
Mitsios et al. [35]	2005	Cohort	51	ACS PCI	G1: A + Clo G2: Clo	5 weeks	PA, P-selectin, CD40L	7
Mukherjee et al. [36]	2005	Prospective cohort	1651	ACS	G1: CYP3A4 + Clo G2: non-CYP3A4 + Clo G3: Clo	6 months	death, MI, stroke, MACE	8
Lau et al. [4]	2003	Prospective cohort	44	PCI	G1: A + Clo G2: P + Clo G3: Clo	24 h	PA	8
Matetzky et al. [37]	2010	Cohort	120	STEMI	G1: statin + Clo G2: Clo	72 h	PA	9
Brophy et al. [16]	2006	Retrospective cohort	2927	PCI	G1: A + Clo G2: Clo	30 days	death, MI, UA, repeat revascularizations, stroke, transient ischemic attack	7
Pelliccia et al. [38]	2014	RCT	122	CAD	G1: A40 + Clo G2: R20 + Clo	30 days	PRU, lipids	–
J.-M.Lablanche et al. [39]	2010	RCT	753	ACS	G1: A80 + Clo G2: R20 + Clo	3 months	lipids, MI, stroke, death, UA, repeat revascularization	–
Guo et al. [40]	2017	RCT	137	ACS PCI	G1: R10 + Clo G2: Clo	1 year	Restenosis, death, MI, target vessel revacularization	–

G: group; RCT: randomized controlled trial; ACS: acute coronary syndromes; CAD: coronary artery disease; MI: myocardial infarction; UA: unstable angina; PCI: percutaneous coronary intervention; PA: platelet aggregation; PRU: platelet reactive unit; LDL-C: low-density lipoprotein cholesterol; HDL-C: high-density lipoprotein cholesterol; TC: total cholesterol; CRP: C-reactive protein; MACE: major adverse cardiovascular events; A: atorvastatin; P: P:pravastatin; R:rosuvastatin; F:fluvastatin; Clo: clopidogrel

**Table 2:** Quality assessment of the randomized controlled trials

Study	Random sequence generation	Allocation concealment	Blinding of participants	Blinding of outcome	Incomplete outcome data addressed	Non-elective reporting	Other bias
Lofti et al. [20] (PROVE-IT)	U	U	L	L	L	L	L
Malmstrom et al. [21]	U	U	U	L	L	L	L
Mitsios et al. [22]	U	U	U	U	L	L	L
Park et al. [24]	U	U	L	L	L	L	L
Poyet et al. [25] (OSCAR)	U	U	U	U	L	L	L
Wenaweser et al. [30]	U	U	L	L	L	U	L
Wenaweser et al. [31]	U	U	U	U	L	L	U
Zhang et al. [32]	L	L	U	U	L	L	L
Pelliccia et al. [38]	U	U	U	U	L	L	U
J.-M.Lablanche et al. [39] (CENTAURUS)	L	L	L	U	L	L	L
Guo et al. [40]	U	U	U	U	L	L	H

*H* high risk, *L* low risk, *U* unclear

## Results

### Literature search and study characteristics

**Fig. 1:**
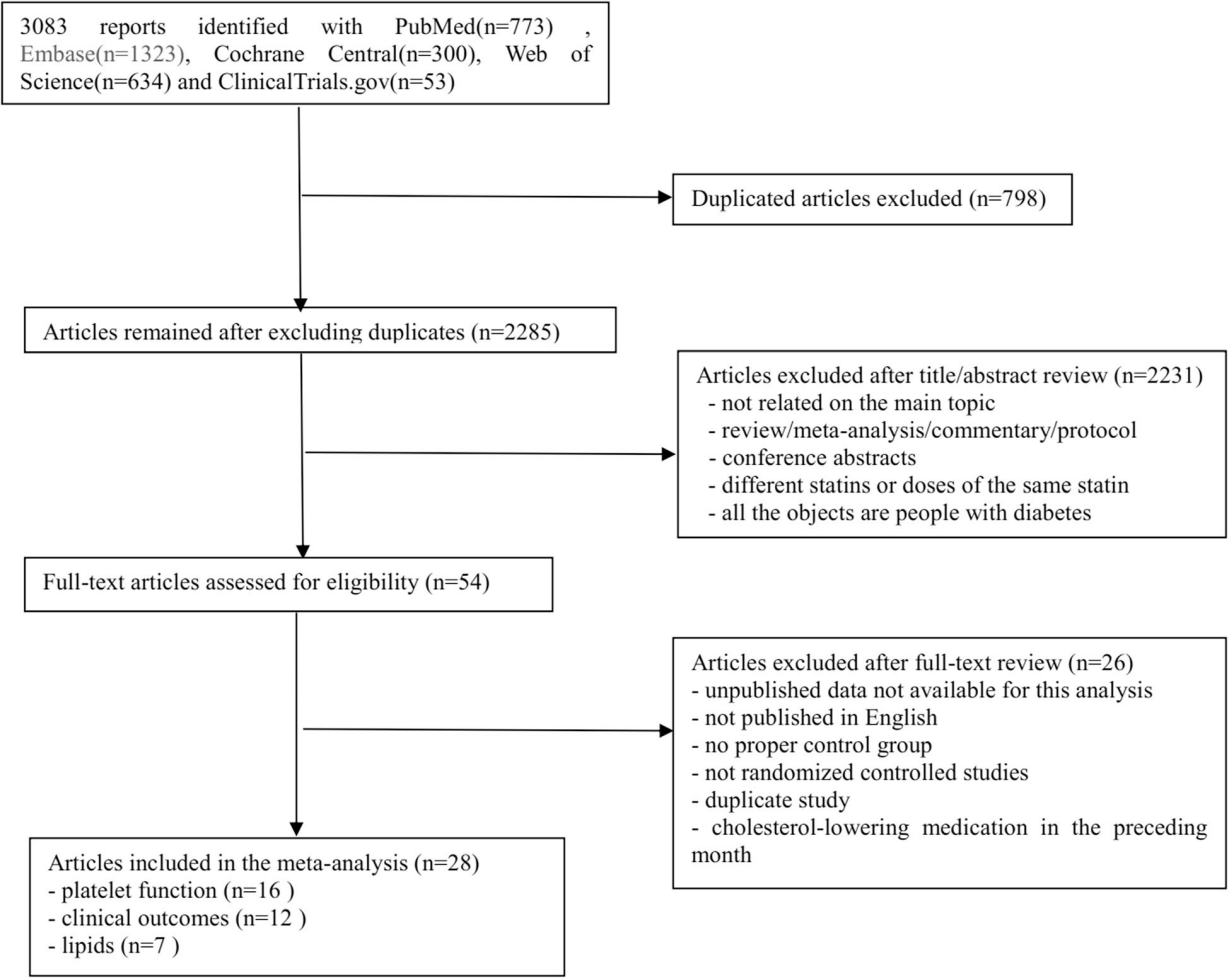
Flow diagram

The literature search results and study selection process are shown in Fig. 1. In total, 3083 potentially suitable articles were identified, where 773 were from PubMed, 1323 from Embase, 634 from Web of Science, 300 from the Cochrane Central Register of Controlled Trials and 53 from ClinicalTrials.gov. The detailed characteristics of these trials are summarized in Table 1. Of these 28 trials, a total of 25,267 participants were included, with sample sizes ranging from 44 to15693 in individual trials.

### Data synthesis and statistical analysis

The meta-analyses and statistical analyses were undertaken using stata11.0. To overcome a unit-of-analysis error, for studies with multiple intervention groups, numbers of groups were proportional distribution. We analyzed outcomes reported at the last available time point when studies reported outcome variables at different time points throughout the intervention period.

Statistical heterogeneity between studies was evaluated using I^2^ statistics, and I^2^ value>50% was defined as heterogeneous. We used Cohen’s to represent the standardized mean difference (SMD) or risk ratio (RR) for each included study because of the use of different measurement techniques to assess platelet indexes, the SMDs and 95% confidence intervals (CIs) for each study were pooled using a random-effects model. Funnel plot and the Egger test were used to test for publication bias. A 2-sided P<0.05 was considered statistically significant. More details of results of partial negative effect, sensitivity analysis and publication bias of included trials were displayed in Additional files 2, 3 and 4.

### Meta-analysis 1: statin + clopidogrel versus clopidogrel

#### Effect on PA indicator

**Fig. 2:**
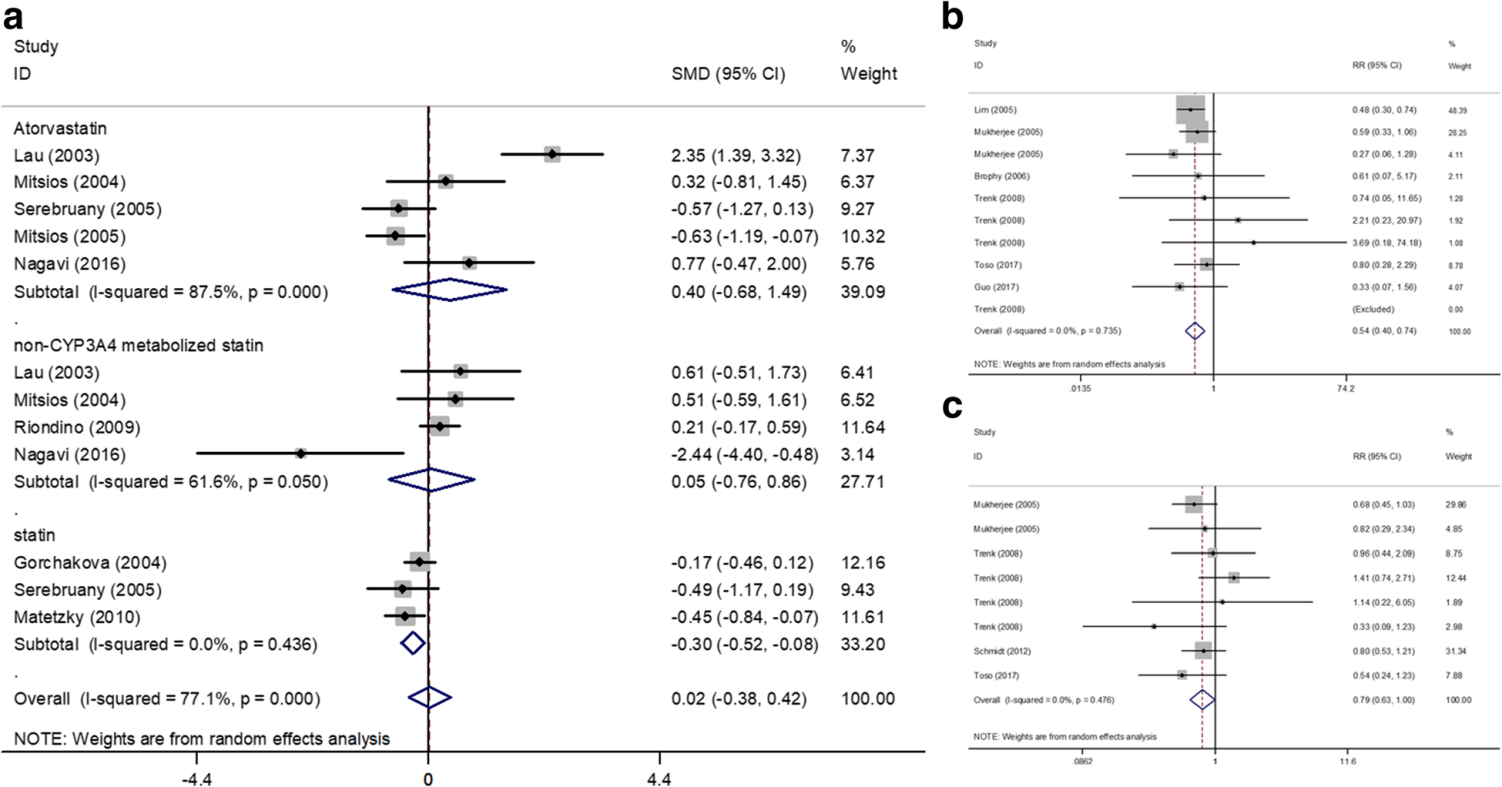
Interaction between statin + clopidogrel and clopidogrel. **a**. The effect on platelet aggregation. **b**. The effect on death. **c**. The effect on major adverse cardiovascular events. CI: confidence interval; RR: risk ratio; SMD: standard mean difference; CYP3A4, cytochrome P450 isoenzyme 3A4

Among the 17 studies comparing the effects of statin and clopidogrel versus clopidogrel, 8 detected data (363 statin group, 273 control group) on PA (Fig. 2a). The superiority of statin plus clopidogrel was not confirmed (SMD 0.02; 95% CI -0.38,0.42; *p* = 0.920) with significant heterogeneity (I^2^ = 77.1%, *p* = 0.000).

#### Effect on residual platelet aggregation (RPA)indicator

Of the 17 trials in the statin plus clopidogrel versus clopidogrel, 2 provided data on the RPA. No significant difference was observed (SMD -0.02; 95% CI -0.10,0.07; *p* = 0.700). No heterogeneity was detected in RPA (I^2^ = 6.8%, *p* = 0.368).

#### Effect on P-selectin(CD62P) indicator

6 trials were identified among the included 17 trials. No reduction was observed in the P-selectin (SMD -0.04; 95% CI -0.14,0.05; *p* = 0.346). However, no heterogeneity was observed in the level of P-selectin (I^2^ = 0.0%, *p* = 0.858).

#### Effect on CD40L, CD63 (LAMP-3),PAC-1 indicators

3 trials detected data on CD40L, while 2 studies provided data on CD63 and PAC-1. No change was found in the following: CD40L (SMD 0.09; 95% CI -0.29,0.48; *p* = 0.633), CD63 (SMD 0.09; 95% CI -0.01,0.19; *p* = 0.079), PAC-1 (SMD 0.03; 95% CI -0.08,0.13; p = 0.633). No heterogeneities were detected in CD40L (I^2^ = 22.1%, *p* = 0.274), CD63 (I^2^ = 0.0%, *p* = 0.916), PAC-1 (I^2^ = 0.0%, *p* = 0.650).

#### Effects on clinical outcomes (including death, MI [myocardial infarction], stroke, MACE[major adverse cardiovascular events])

Death event was recorded in the 6 trials (Fig. 2b). The benefit of clopidogrel was significantly influenced of concomitant treatment with a statin and this was irrespective of treatment with CYP3A4 metabolized statin (RR 0.54; 95% CI 0.40,0.74; *p* = 0.000) with no heterogeneity (I^2^ = 0.0%, *p* = 0.735).

5 trials with 5346 participants provided the incidence of MI. The pooled estimates of trials demonstrated that no difference was found (RR 1.0; 95% CI 0.67,1.48; *p* = 0.994) with no heterogeneity (I^2^ = 0.0%, *p* = 0.675).

Stroke was reported in 5 studies with 1 trial missing data. The combined therapy failed to reduce the occurrence of stroke (RR 0.98; 95% CI 0.60,1.60; *p* = 0.944). Heterogeneity was not observed (I^2^ = 3.4%, *p* = 0.376).

MACE was identified in 4 trials (Fig. 2c). No difference was found (RR 0.79; 95% CI 0.63,1.00; *p* = 0.047) with no heterogeneity (I^2^ = 0.0%, *p* = 0.476).

### Meta-analysis 2: CYP3A4 metabolized statin + clopidogrel versus non-CYP3A4 metabolized statin + clopidogrel

#### Effect on PA indicator

**Fig. 3:**
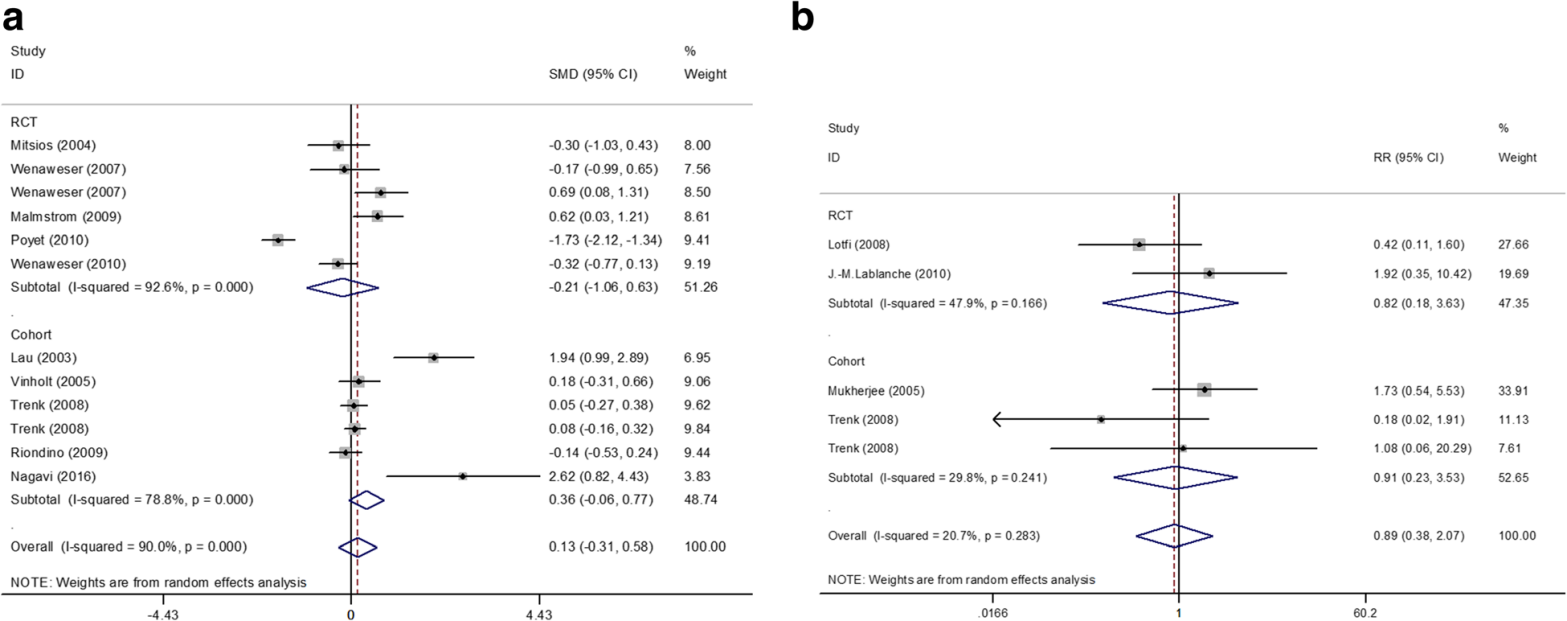
Interaction between CYP3A4 statin + clopidogrel and non-CYP3A4 statin + clopidogrel **a**. The effect on platelet aggregation. **b**. The effect on death. CI: confidence interval; SMD: standard mean difference; RCT, randomized controlled trial

A total of 10 clinical studies involving 1279 participants 874 CYP3A4 metabolized, 405 non-CYP3A4 metabolized were included in the analysis to investigate the effect of different type of statin (Fig. 3a). The overall pooled mean difference on PA was 0.13 (SMD 0.13; 95% CI -0.31,0.58; *p* = 0.556). However, different research types showed high heterogeneity (I^2^ = 90.0%, *p* = 0.000).

#### Effect on P-selectin(CD62P) indicator

A total of 4 trials with 5 comparisons involved 840 patients (651 assigned to CYP3A4 metabolized statin therapy, 189 assigned to non-CYP3A4 metabolized statin therapy) reported the effect on P-selectin. The overall pooled mean difference on P-selectin was 0.17 (SMD 0.17; 95% CI -0.16,0.51; *p* = 0.310) with low heterogeneity (I^2^ = 65.8%, *p* = 0.020). However, RCTs showed significant difference on P-selectin (SMD 0.52; 95% CI -0.17,0.88; *p* = 0.004), while cohort study showed no difference (SMD -0.13; 95% CI -0.33,0.06; *p* = 0.186).

#### Effect on lipid metabolism indicators

Among the 7 studies comparing the effects of lipids, 6, 5, 5 and 4 detected data on the changes in LDL-C, HDL-C and TC level respectively. No reduction was observed in the following: LDL-C (SMD 0.02; 95% CI -0.35,0.40; *p* = 0.900), HDL-C (SMD -0.55; 95% CI -1.15,0.04; *p* = 0.069), TC (SMD -0.04; 95% CI –0.25,0.17; *p* = 0.723). Compared to non-CYP3A4 metabolized statin, the CYP3A4 metabolized statin remarkably decreased the TG level (SMD -0.19; 95% CI –0.33,-0.06; *p* = 0.005) with no heterogeneity (I^2^ = 24.1%, *p* = 0.267) (Fig. 3b). Heterogeneities were detected in LDL-C (I^2^ = 90.3%, *p* = 0.000), HDL-C (I^2^ = 96.1%, p = 0.000), HDL-C (I^2^ = 96.1%, p = 0.000), TC (I^2^ = 64.6%, *p* = 0.023).

#### Effect on clinical outcomes(including death, MI, stroke and MACE)

The incidence of death was reported in 5 studies with 1 missing data. Compared to non-CYP3A4 metabolized statins, CYP3A4 metabolized statins did not reduce mortality (RR 0.89; 95% CI 0.38,2.07; *p* = 0.791) with no heterogeneity (I^2^ = 20.7%, *p* = 0.283).

MI data were provided in 5 studies with 1 missing data. The pooled effect demonstrated that no difference was found (RR 0.82; 95% CI 0.52,1.28; *p* = 0.377) with no heterogeneity(I^2^ = 0.0%, *p* = 0.575).

Stroke was reported in 3 studies. Compared to non-CYP3A4 metabolized statin plus clopidogrel, the CYP-3A4 metabolized statin therapy combined clopidogrel failed to reduce the incidence of stroke (RR 0.86; 95% CI 0.13,5.61; *p* = 0.875).

MACE was recorded in 4 trials. The pooled effect was found no difference (RR 0.95; 95% CI 0.68,1.34; *p* = 0.788).

## Discussion

Our meta-analysis revealed that in patients receiving concomitantly statin and clopidogrel, statins significantly reduced mortality and MACE without affecting platelet activation and aggregation. Furthermore, CYP3A4 metabolized and non-CYP3A4 metabolized statins had similar influence on platelet activity, lipid metabolism and clinical outcomes, except for TG level. CYP3A4 metabolized statins more greatly reduced the level of TG than CYP3A4 metabolized statins.

The effect of combination of statin plus clopidogrel on platelet activity have aroused people’s concern. Lau et al. first reported the negative interference of atorvastatin with the antiplatelet effect of clopidogrel [6]. And some other trials also drove the same result [22, 34, 35]. In contrast, Serebruany et al. showed no difference in platelet inhibitory effects of clopidogrel in patients undergoing coronary stent placement taking statins [10]. And other studies [8, 12, 33, 37, 41–43], in accordance with our results, also showed neutral effects of statin on platelet inhibition by clopidogrel. Even more, the main finding of ACHIDO (Atorvastatin and Clopidogrel High DOse in stable patients with residual high platelet activity) study was that high-dose statin improved the pharmacodynamic effects of 150 mg clopidogrel [44]. Possible explanations for this discrepancy could be attributed to the small sample size, lack of strict inclusion criteria, indirect methods for assessment of platelet function and different responses to clopidogrel among patients. Patients with diabetes were often low responders to clopidogrel [21, 45, 46].

Our meta-analysis not only compared the antiplatelet efficacy of statin plus clopidogrel, but also detected the difference between CYP3A4 metabolized statin and non-CYP3A4 metabolized statin on PA parameters. But the comparison between two groups failed to find statistical difference. A prospective, randomized study also concluded the same result that no difference was found between atorvastatin and fluvastatin treatment arms [47]. And the result was in accordance with some other trials [30, 42, 48, 49]. However, Neubauer et al. concluded that simvastatin and atorvastatin appear to significantly inhibit the activation of clopidogrel after administration of a loading dose, which reduced by 29.3% at 5 h, 16.6% at 48 h. And some related researches in healthy people also drove the same conclusion [50, 51]. Increasing evidence represented no potential influence of statin on the antiaggregatory effect of clopidogrel, regardless of the type of statins. Several studies assessed RPA also drove the same result that residual ADP-induced platelet aggregation was not significantly different between statin-treatment group and statin naïve group, neither CYP3A4 metabolized statin nor non-CYP3A4 metabolized statin [13, 21, 37].To reflect the multiple effects of P2Y12 receptor activation on platelet responses, platelet activation markers like granular membrane protein 140 (P-selectin), human soluble cluster of differentiation 40 ligand (sCD40L), CD63 (LAMP-3), PAC-1 were also measured. P-selectin [32], a glycoprotein located in blood platelets and endothelial cells, is a specific molecular marker for platelet activation. When platelets are activated, the P-selectin concentrations on the platelet membranes in plasma are increased. CD40L is a type II membrane protein-transporting molecule expressed by active platelet. After activation, CD40L is released into blood in a soluble manner. In general, no statistical difference was observed when patients administered with statin and clopidogrel, regardless of types of statin. For one thing, clopidogrel can be converted to active metabolites by many isozymes, including CYP 3A4, CYP3A5, CYP1A, CYP2B. For another, the plasma concentration of statin is not enough to reach the saturation of enzyme concentration to cause competitive inhibition. What is more, the expression of inflammatory mediators in ACS is higher than non-ACS, which could promote platelet aggregation. However, statins have a strong anti-inflammatory effect. Even with competitive inhibition, it may be compensated by the anti-inflammatory effect of statin [52]. Finally, it should not be ignored that so many factors can weaken curative effect of clopidogrel, like gene polymorphism, hyperinsulinemia or insulin resistance [27, 53–55].

We also detected the interaction between statin and clopidogrel on the aspect of lipid levels, and no statistic influence on lipids level was observed, except for TG level. Four randomized and placebo-controlled trials [20, 25, 38, 39] compared lipid variables in LDL-C and HDL-C between atorvastatin and rosuvastatin and no differences were found. Serebruany et al. general described that TC levels were lower in patients treated with a statin than in those receiving no statin [10]. It is a pity that few articles did deeply into the specific lipid-lowering effect of clopidogrel on statin. Thus, more studies regarding the effects of the lipid parameters were needed to determine whether clopidogrel can decrease lipid levels to perfect the interaction between clopidogrel and statins.

In addition, our study elaborated the impact of combined statin and clopidogrel on clinical outcomes and found that the incidence of death was remarkably decreased. However, other adverse events including stroke, MI were not decreased. Our finding is in accordance with the PROVE IT-TIMI 22 (Pravastatin or Atorvastatin Evaluation and Infection Therapy-Thrombolysis in Myocardial Infarction 22) trial which showed that statin therapy ameliorate long-term clinical events, whether patients taking atorvastatin or pravastatin, which included >72% did receive clopidogrel, although clopidogrel was not mandated [56]. Our data also concur with GRACE (Global Registry of Acute Coronary Events), MITRA PLUS (Maximal Individual Therapy of Acute Myocardial Infarction PLUS) and TRITON-TIMI 38 (Trial to Assess Improvement in Therapeutic Outcomes by Optimizing Platelet Inhibition With Prasugrel-Thrombolysis In Myocardial Infarction 38)., suggesting that the combination of clopidogrel with a stain was not related to an increased risk of end points [11, 16, 24]. One crude cohort study reported that no difference was found in the composite of death, MI, any revascularization [57]. And another cohort study also indicated that statin was not associated with an increased early risk of adverse cardiovascular events [36]. From above, additional statin should inevitably be used to obtain better clinical benefits. And our result showed that the treatment of clopidogrel with CYP3A4 metabolized or non-CYP3A4 metabolized statin has no specific difference. A post-hoc analysis of the CREDO trial, reported that statin combined with clopidogrel significantly reduced cardiovascular events compared with clopidogrel alone [14, 15]. The adverse clinical effect did not differ between the group using clopidogrel either with a statin metabolized by CYP3A4 or a statin not metabolized through CYP3A4. In parallel with our meta-analysis, Mukherjee et al. reported that no statistically significant difference was noted in MACE, stroke, MI, or death at 6 months between those receiving a CYP3A4 metabolized or non-CYP3A4 metabolized statin with clopidogrel therapy [26]. CHARISMA (Clopidogrel for High Atherothrombotic Risk and Ischemic Stabilization, Management, and Avoidance), a large randomized prospective trial which includes 15,574 patients, found that long-term concomitant clopidogrel and statin therapy was associated with a lower primary efficacy end points (MI, stroke, or cardiovascular death)As can be seen from above studies, these studies are different in study design and are not reasonable. The number of cases treated with a single type of statin is still small and the follow-up time is relatively short. However, these results most suggest that combination of statin and clopidogrel should be applied to maximize the cardiovascular benefits, regardless of the statin type, for the reason that the advantage outweigh the disadvantage.

This meta-analysis has several strengths. First, it is to date the most comprehensive analysis that systematically and quantitatively assess the correlation between statins and clopidogrel. Second, indexes to assess the progression of platelet activation and aggregation are taken into consideration. Third, lipid metabolism levels are enriched in this meta-analysis. Furthermore, many clinical outcomes are considered.

This meta-analysis also has several limitations. First, as with any meta-analysis, the internal validity depends on the methodological quality of the included studies. There is unfortunately no one ideal test that will directly indicate the in vivo antiplatelet effect and predict the clinical consequences. Second, the duration of follow-up varied among the included patients and heterogeneities existed among trials. Third, despite no significant publication bias was detected by the Begg’s test and Egger’s test for each result, the risk of publication bias still cannot be fully ruled out due to the language restriction to English. Additionally, the absence of standardization in study design, characteristics of the study populations were not uniformed. Last but not least, Impaired response to antiplatelet therapy in diabetic patients has been reported. Thus, further prospective studies are warranted to clarify the potential interactions between clopidogrel and statins and to determine whether a true clinical effect exists.

## Conclusion

Statins reduce mortality in patients undergoing clopidogrel treatment without affecting platelet activation and aggregation, either with CYP-3A4 metabolized statin or non-CYP3A4 metabolized statin. However, the level of TG is reduced when people administered with clopidogrel and CYP3A4 metabolized statin, compared to non-CYP3A4 metabolized statin. The effect of clopidogrel on lipid parameters and clinical outcomes of people receiving statin is still unknown. Further researches are needed to elucidate the mutual interaction between statin and clopidogrel on more aspects.

## Additional file

Additional file 1: Search strategy of PubMed. (DOCX 17 kb)

Additional file 2: Results of sensitivity analysis. (DOCX 11093 kb)

Additional file 3: Results of publication bias of included trials. (DOCX 26703 kb)

Additional file 4: Contents. (DOCX 34172 kb)

## Abbreviations

ACS: acute coronary syndrome
CAD: coronary artery disease
CI: Confidence interval
CYP3A4: cytochrome P450 isoenzyme 3A4
HDL-C: high-density lipoprotein cholesterol
LDL-C: low-density lipoprotein cholesterol
MACE: major adverse cardiovascular events
MI: myocardial infarction
PA: platelet aggregation
RCT: randomized controlled trial
RR: risk ratio
SMD: standard mean difference
TC: total cholesterol
TG: triglycerides
