# Supplementary material for: Statins significantly reduce mortality in patients receiving clopidogrel without affecting platelet activation and aggregation: a systematic review and meta-analysis
Source: Lipids Health Dis. 2019 May 24;18:121. doi: 10.1186/s12944-019-1053-0 (PMC6533696; doi:10.1186/s12944-019-1053-0)
Supplement: Supplementary file 1 — Search strategy of PubMed. (DOCX 17 kb) [file 12944_2019_1053_MOESM1_ESM.docx]

**Additional file 1:** Search strategy of PubMed

| PubMed | | |
| --- | --- | --- |
| #1 | clopidogrel | 12969 |
| #2 | ((((((((clopidogrel napadisilate[Title/Abstract]) OR clopidogrel besylate[Title/Abstract]) OR clopidogrel besilate[Title/Abstract]) OR clopidogrel hydrochloride[Title/Abstract]) OR clopidogrel Sandoz[Title/Abstract]) OR clopidogrel bisulfate[Title/Abstract]) OR Plavix[Title/Abstract]) OR Platelet Aggregation Inhibitors[Title/Abstract]) OR Purinergic P2Y Receptor Antagonists[Title/Abstract] | 1090 |
| #3 | #1 AND #2 | 13587 |
| #4 | statins | 47298 |
| #5 | ((((((((((((((((((((((Hydroxymethylglutaryl CoA Reductase Inhibitors[Title/Abstract]) OR Inhibitors, Hydroxymethylglutaryl-CoA Reductase[Title/Abstract]) OR Reductase Inhibitors, Hydroxymethylglutaryl-CoA[Title/Abstract]) OR Inhibitors, HMG-CoA Reductase[Title/Abstract]) OR Inhibitors, HMG CoA Reductase[Title/Abstract]) OR Reductase Inhibitors, HMG-CoA[Title/Abstract]) OR HMG-CoA Reductase Inhibitors[Title/Abstract]) OR HMG CoA Reductase Inhibitors[Title/Abstract]) OR Statins, HMG-CoA[Title/Abstract]) OR HMG-CoA Statins[Title/Abstract]) OR Statins, HMG CoA[Title/Abstract]) OR Inhibitors, Hydroxymethylglutaryl-CoA[Title/Abstract]) OR Hydroxymethylglutaryl-CoA Inhibitors[Title/Abstract]) OR Inhibitors, Hydroxymethylglutaryl CoA[Title/Abstract]) OR Inhibitors, Hydroxymethylglutaryl-Coenzyme A[Title/Abstract]) OR Hydroxymethylglutaryl-Coenzyme A Inhibitors[Title/Abstract]) OR Inhibitors, Hydroxymethylglutaryl Coenzyme A[Title/Abstract]) OR atorvastatin[Title/Abstract]) OR lovastatin[Title/Abstract]) OR simvastatin[Title/Abstract]) OR fluvastatin[Title/Abstract]) OR rosuvastatin[Title/Abstract]) OR pravastatin[Title/Abstract] | 27576 |
| #6 | #4 OR #5 | 51465 |
| #7 | #3 AND #6 | 773 |
