# Supplementary material for: Statins significantly reduce mortality in patients receiving clopidogrel without affecting platelet activation and aggregation: a systematic review and meta-analysis
Source: Lipids Health Dis. 2019 May 24;18:121. doi: 10.1186/s12944-019-1053-0 (PMC6533696; doi:10.1186/s12944-019-1053-0)

**Additional file 2:** Results of sensitivity analysis

**Meta-analysis 1: statin + clopidogrel versus clopidogrel**

**Effect on PA indicator**


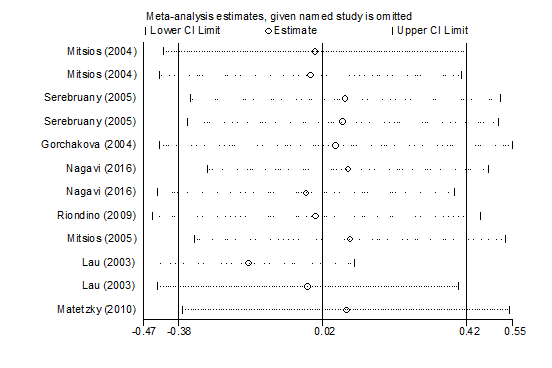


**Effect on RPA indicator**


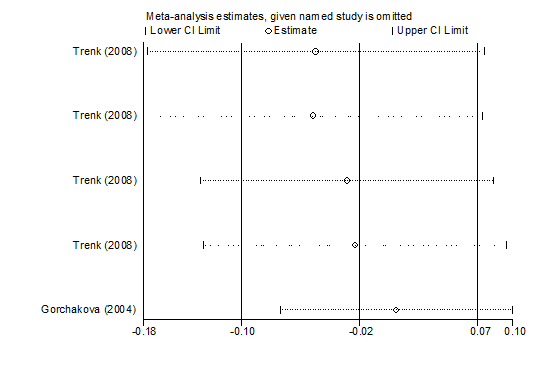


**Effect on P-selectin(CD62P) indicator**


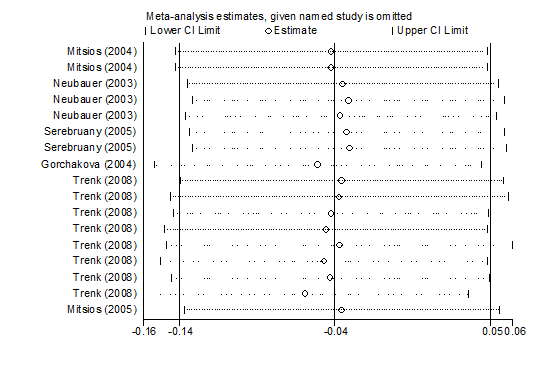


**Effect on CD40L, CD63 (LAMP-3) ,PAC-1 indicators**


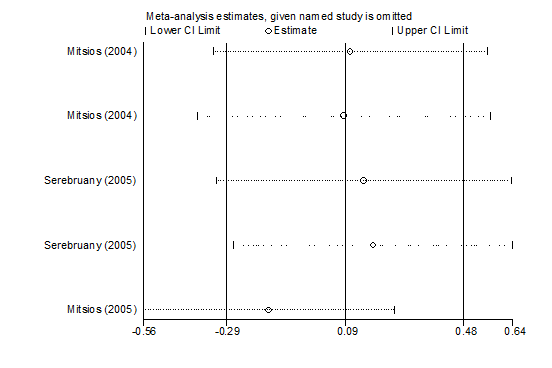


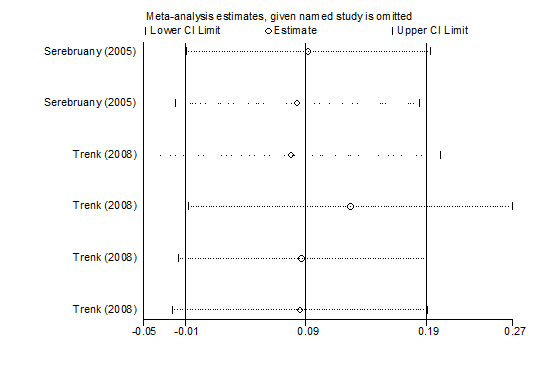


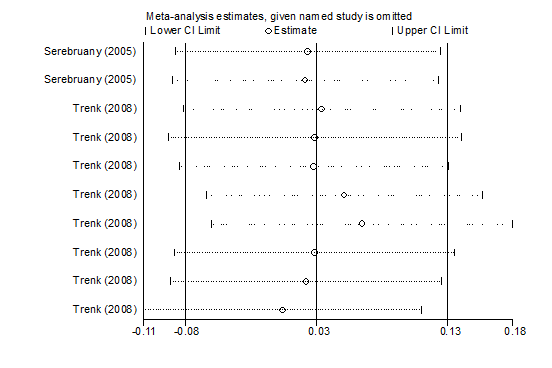


**Effects on clinical outcomes (including death, MI [myocardial infarction], stroke, MACE[major adverse cardiovascular events])**


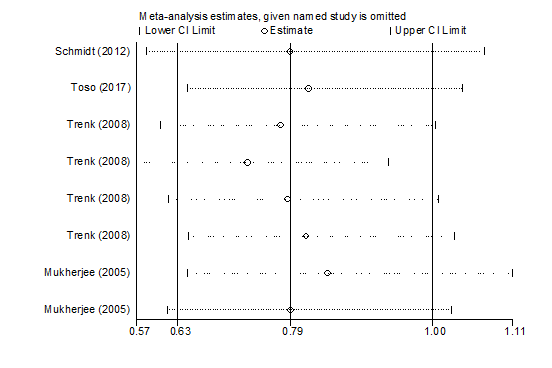


**Meta-analysis 2: CYP3A4 statin + clopidogrel versus non-CYP3A4 statin + clopidogrel**

**Effect on PA indicator**


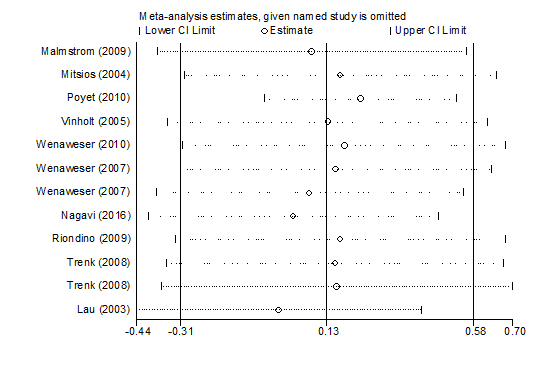


**Effect on P-selectin(CD62P)**


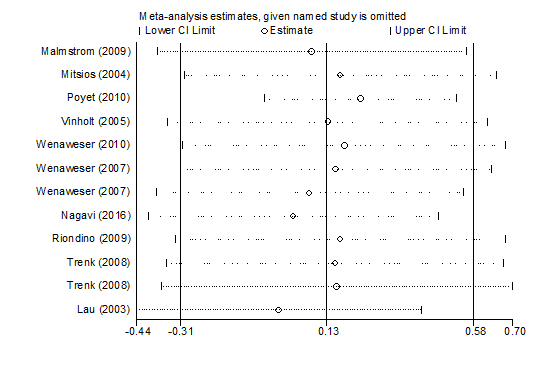


**Effect on lipid metabolism indicators**


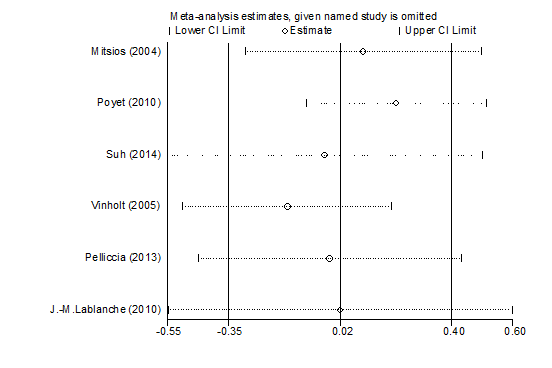


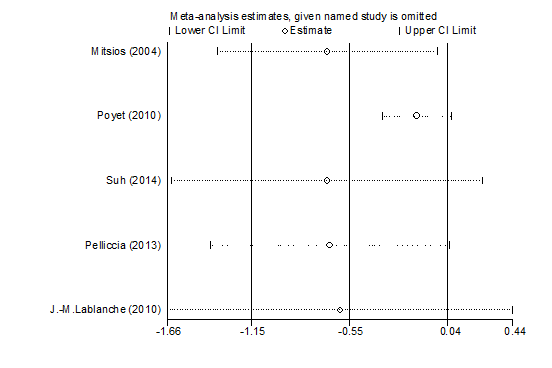


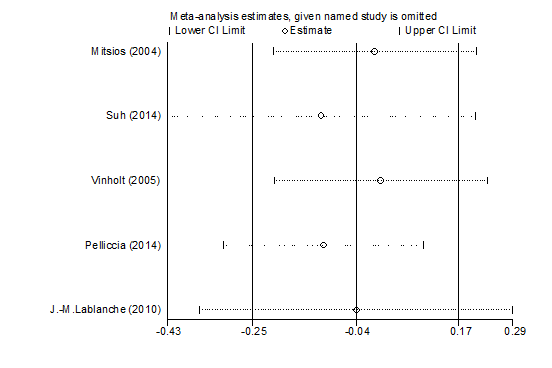


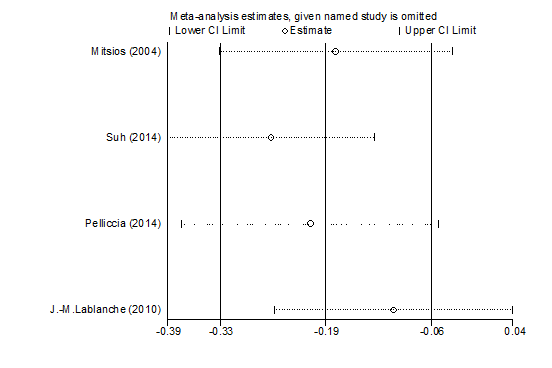


**Effect on clinical outcomes(including death, MI, stroke and MACE)**


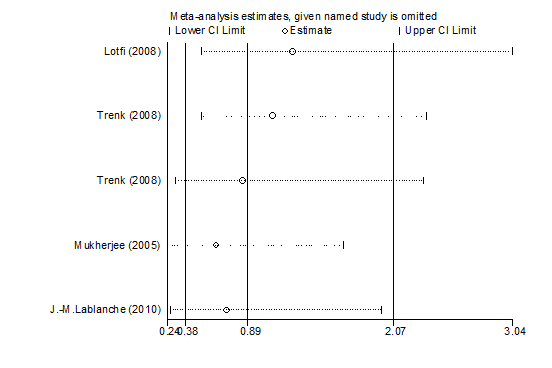


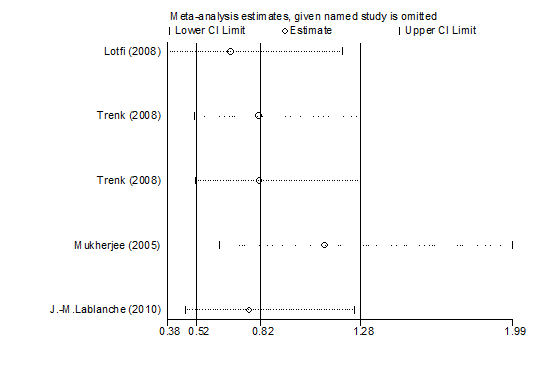


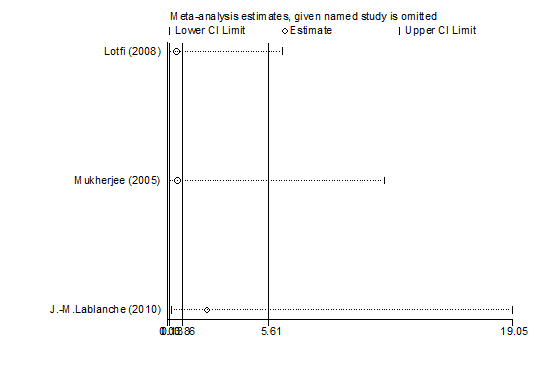


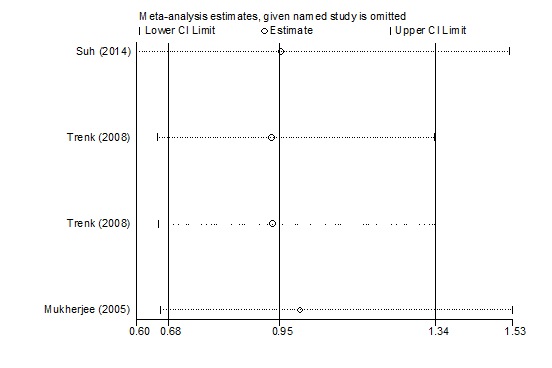

Supplement: Supplementary file 2 — Results of sensitivity analysis. (DOCX 11093 kb) [file 12944_2019_1053_MOESM2_ESM.docx]
