# Supplementary material for: Statins significantly reduce mortality in patients receiving clopidogrel without affecting platelet activation and aggregation: a systematic review and meta-analysis
Source: Lipids Health Dis. 2019 May 24;18:121. doi: 10.1186/s12944-019-1053-0 (PMC6533696; doi:10.1186/s12944-019-1053-0)

**Additional file 3:** Results of publication bias of included trials

**Meta-analysis 1: statin + clopidogrel versus clopidogrel**

**Effect on PA indicator**


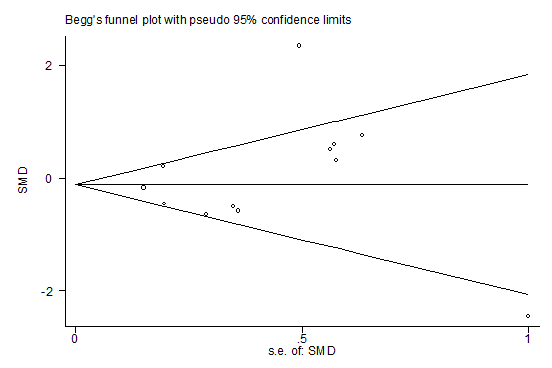


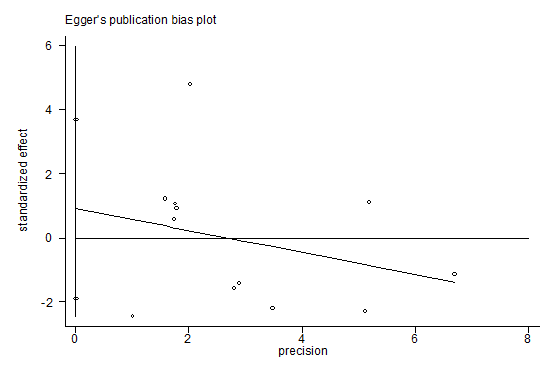


**Effect on RPA indicator**


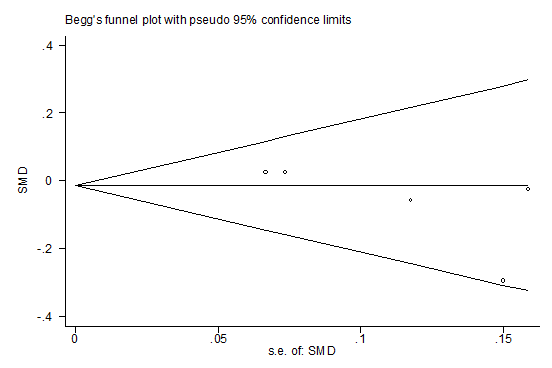


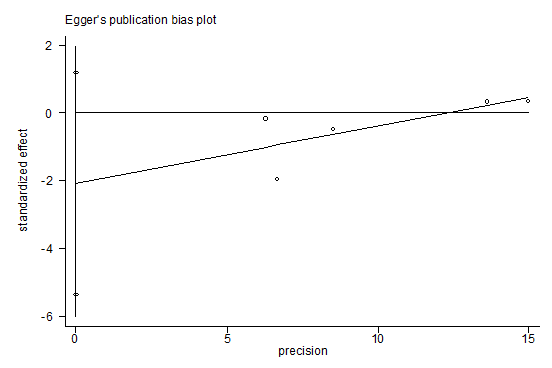


**Effect on P-selectin(CD62P) indicator**


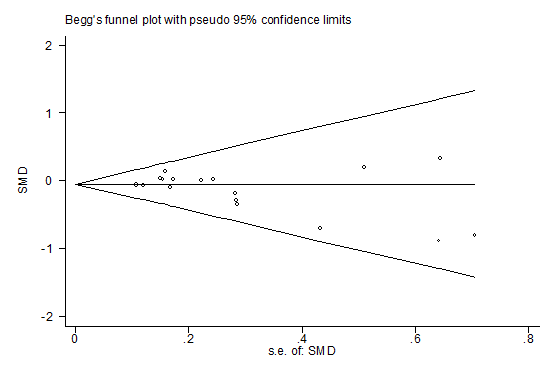


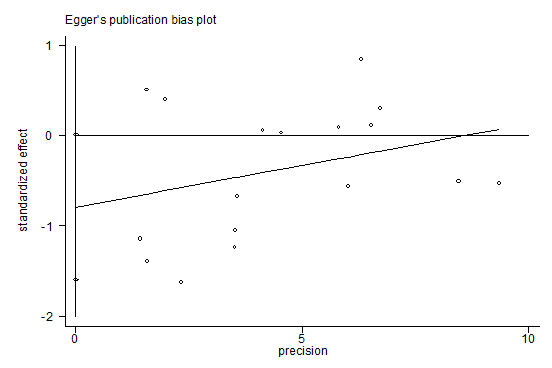


**Effect on CD40L, CD63 (LAMP-3) ,PAC-1 indicators**


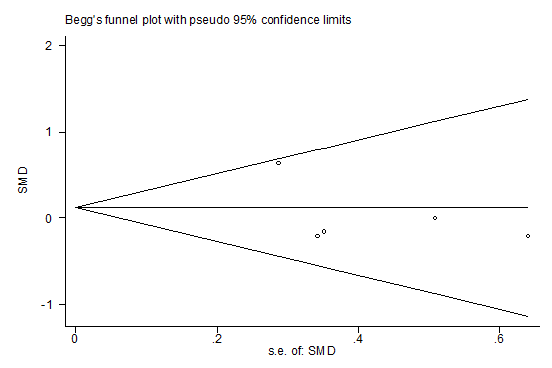


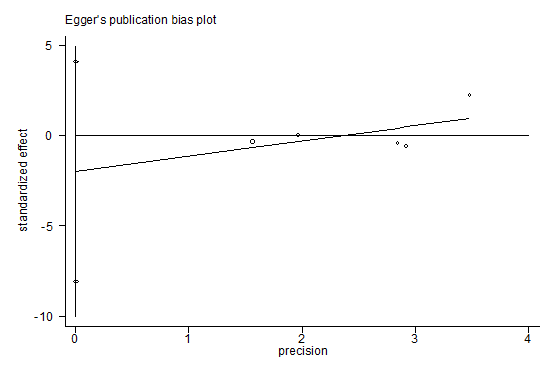


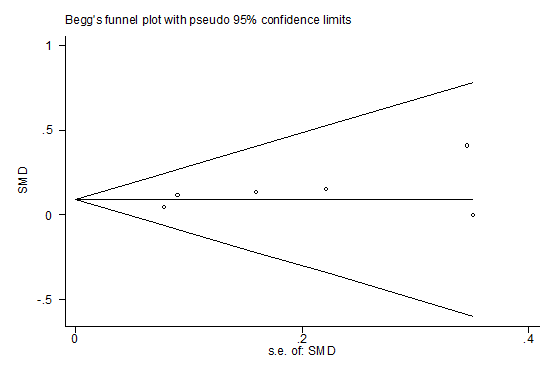


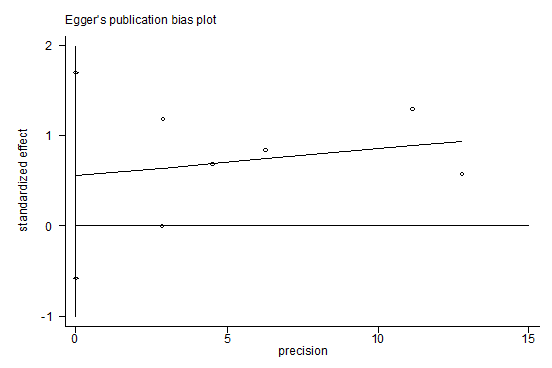


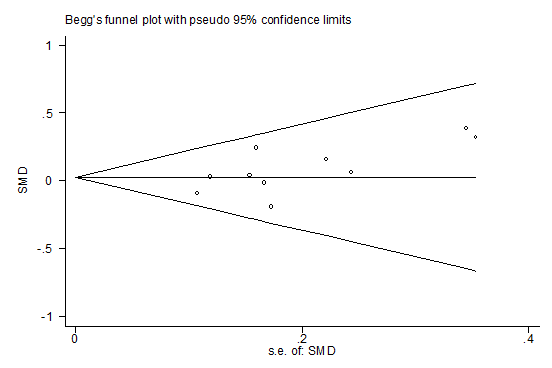


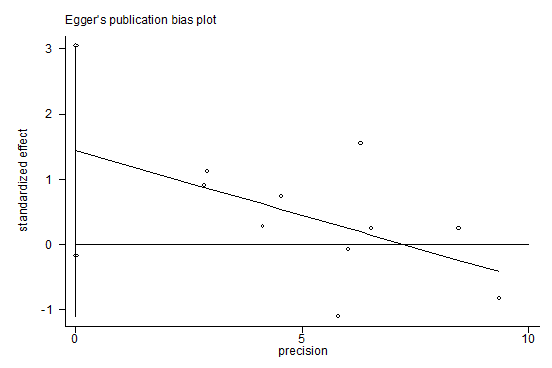


**Effects on clinical outcomes (including death,** **MI [myocardial infarction], stroke, MACE[major adverse cardiovascular events])**


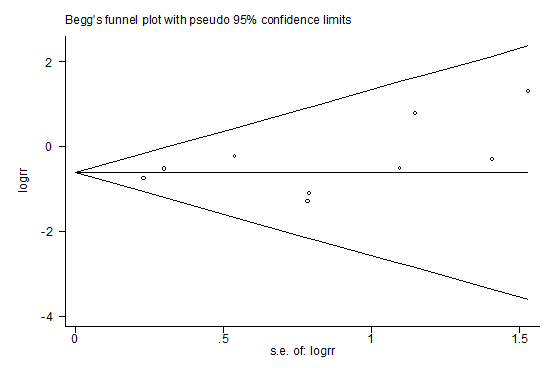


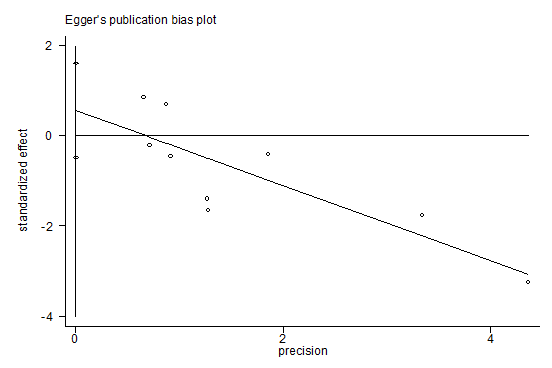


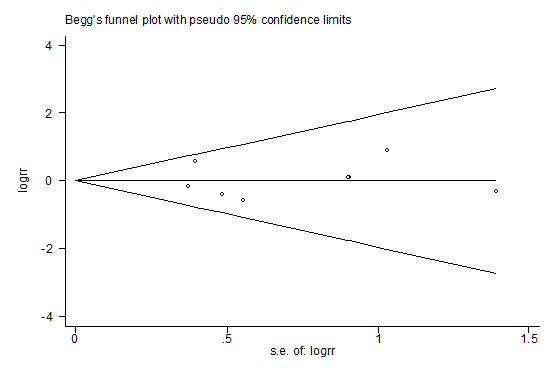


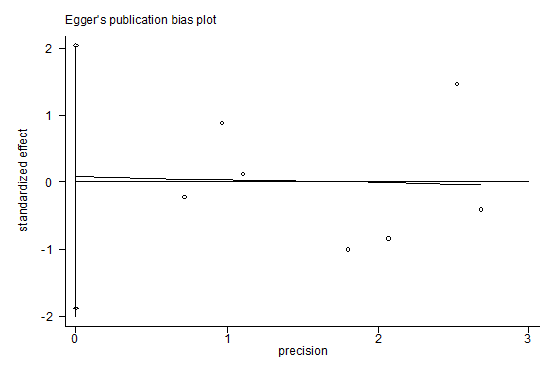


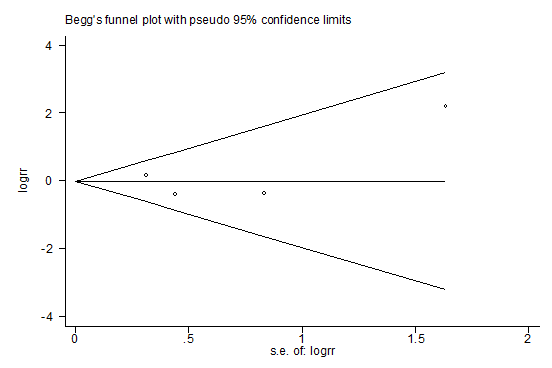


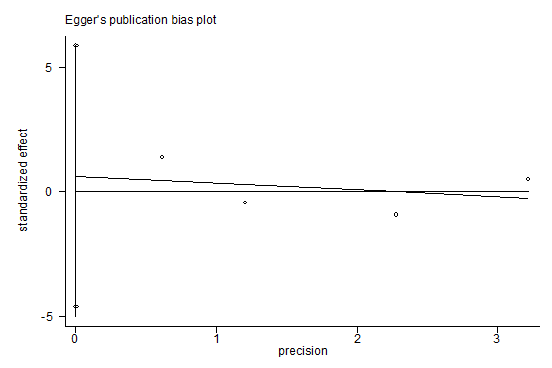


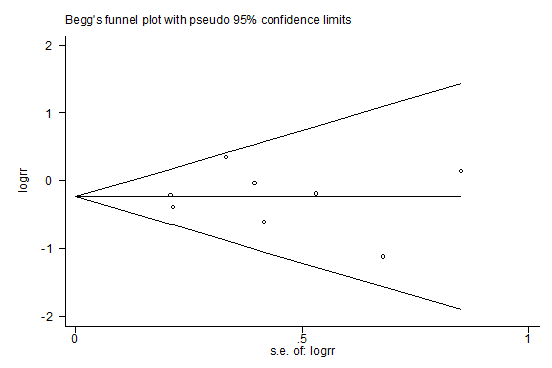


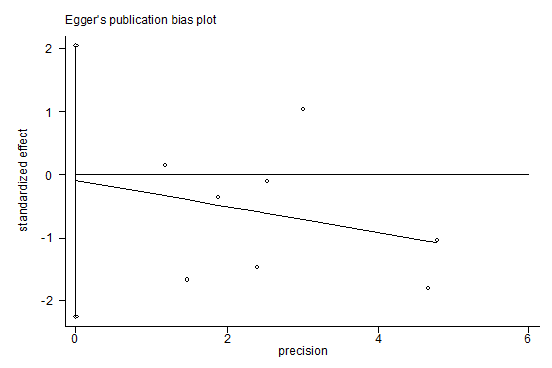


**Meta-analysis 2:** **CYP3A4 statin + clopidogrel versus non-CYP3A4 statin + clopidogrel**

**Effect on PA indicator**


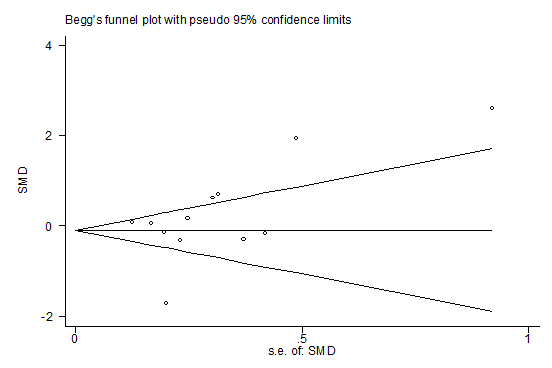


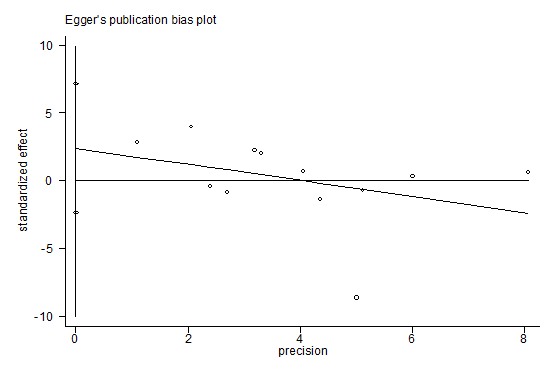


**Effect on P-selectin(CD62P)**


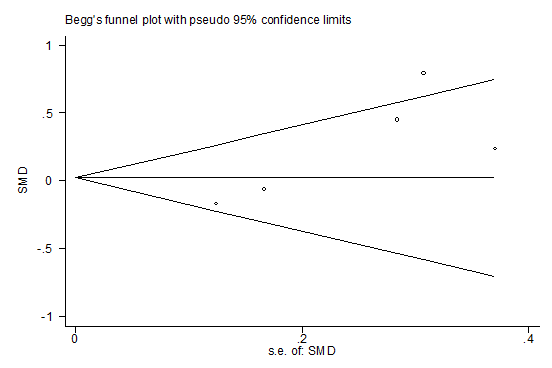


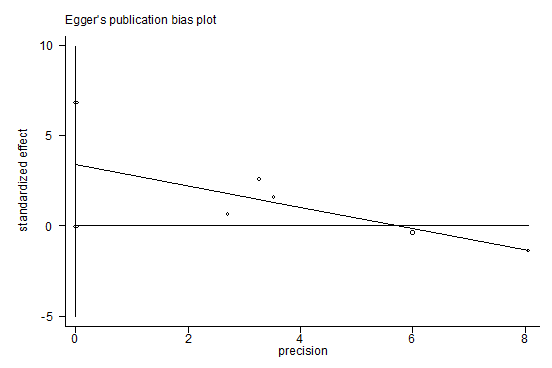


**Effect on lipid metabolism indicators**


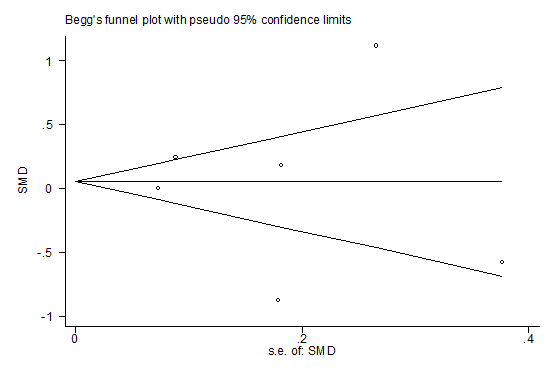


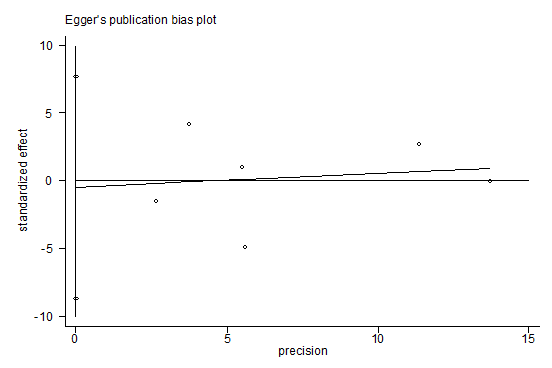


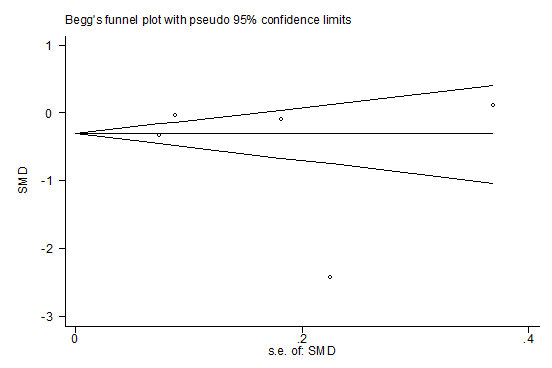


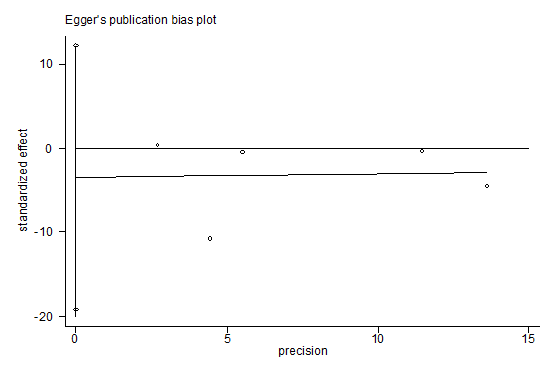


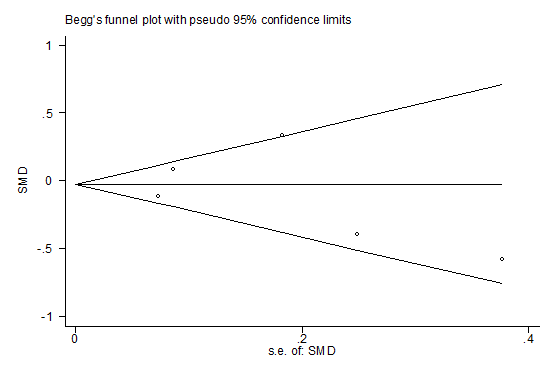


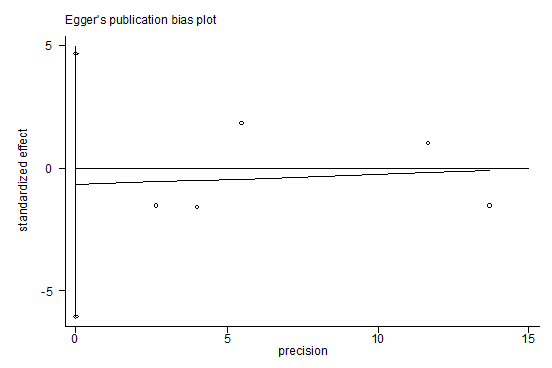


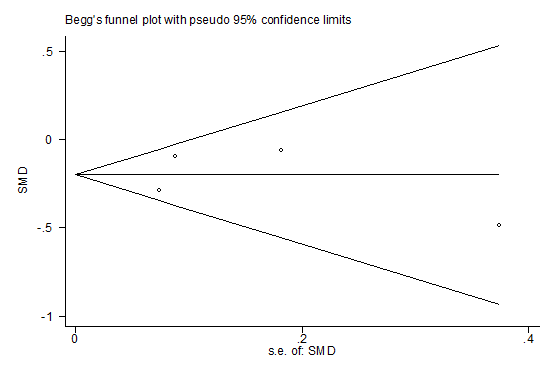


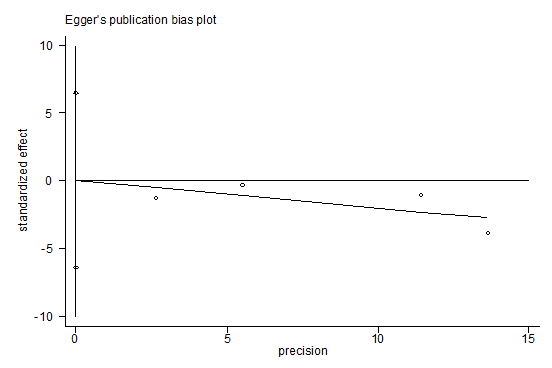


**Effect on clinical outcomes(including death, MI, stroke and MACE)**


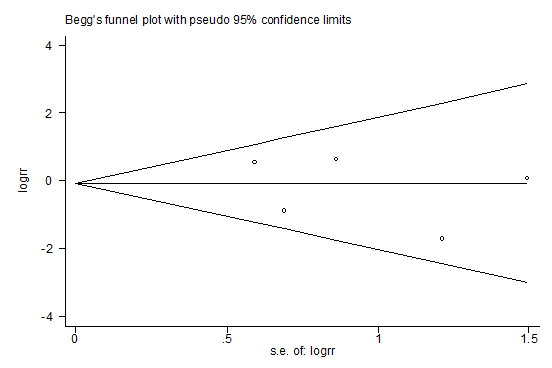


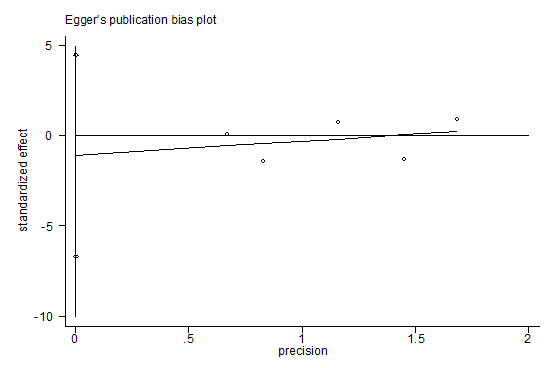


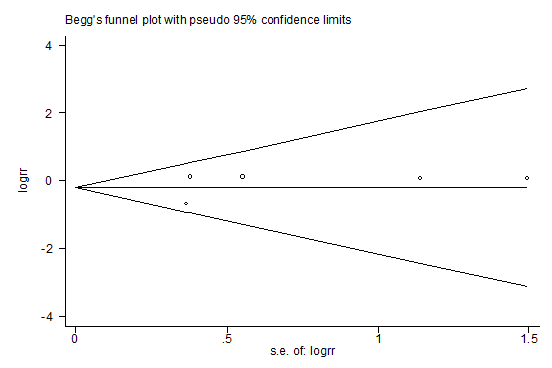


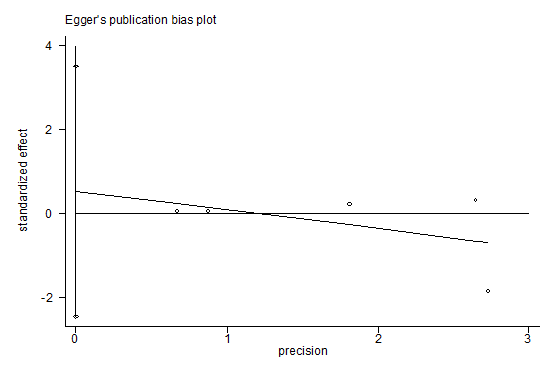


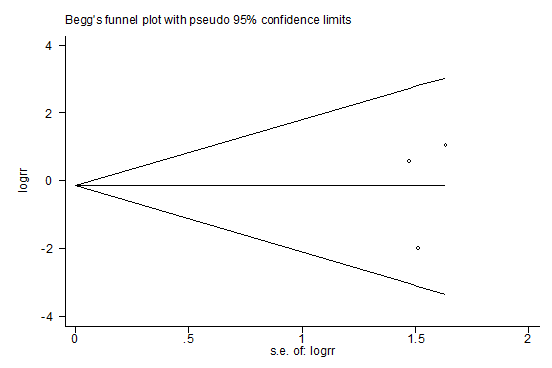


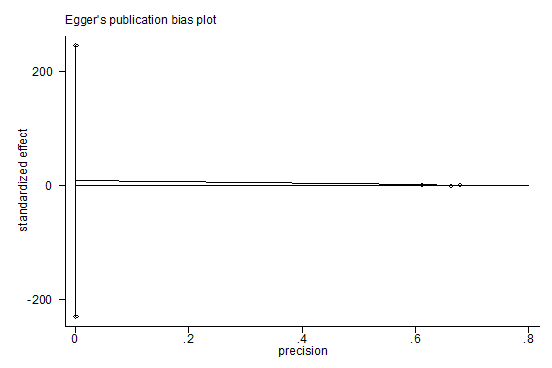


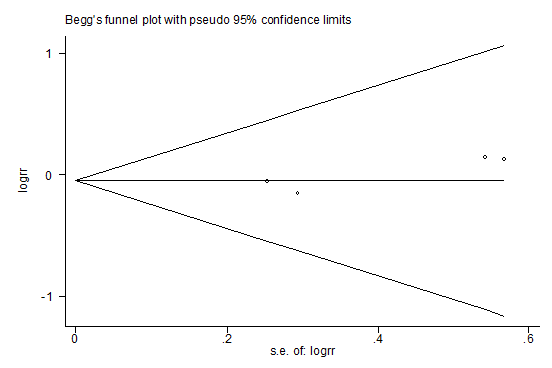


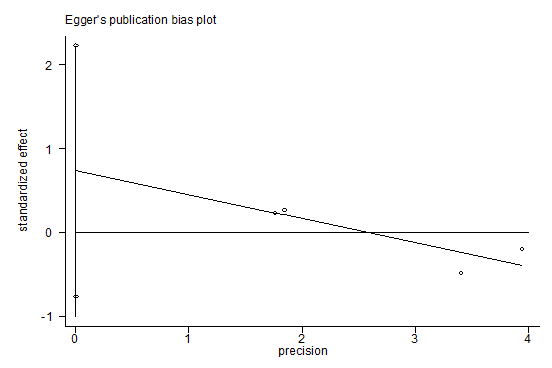

Supplement: Supplementary file 3 — Results of publication bias of included trials. (DOCX 26703 kb) [file 12944_2019_1053_MOESM3_ESM.docx]
