# Supplementary material for: Statins significantly reduce mortality in patients receiving clopidogrel without affecting platelet activation and aggregation: a systematic review and meta-analysis
Source: Lipids Health Dis. 2019 May 24;18:121. doi: 10.1186/s12944-019-1053-0 (PMC6533696; doi:10.1186/s12944-019-1053-0)
Supplement: Supplementary file 4 — Contents. (DOCX 34172 kb) [file 12944_2019_1053_MOESM4_ESM.docx]

**Additional file 4:** Contents

Figure S1. Effect on residual platelet aggregation indicator between statin + clopidogrel and clopidogrel (CYP3A4, cytochrome P450 isoenzyme 3A4)

Figure S2. Effect on P-selectin between statin + clopidogrel and clopidogrel (CYP3A4, cytochrome P450 isoenzyme 3A4)

Figure S3. Effect on CD40L between statin + clopidogrel and clopidogrel (CYP3A4, cytochrome P450 isoenzyme 3A4)

Figure S4. Effect on CD63 (LAMP-3) between statin + clopidogrel and clopidogrel (CYP3A4, cytochrome P450 isoenzyme 3A4)

Figure S5. Effect on PAC-1 between statin + clopidogrel and clopidogrel (CYP3A4, cytochrome P450 isoenzyme 3A4)

Figure S6. Effect on myocardial infarction between statin + clopidogrel and clopidogrel (CYP3A4, cytochrome P450 isoenzyme 3A4)

Figure S7. Effect on stroke between statin + clopidogrel and clopidogrel (CYP3A4, cytochrome P450 isoenzyme 3A4)

Figure S8. Effect on P-selectin between CYP3A4 statin + clopidogrel and non-CYP3A4 statin + clopidogrel (CYP3A4, cytochrome P450 isoenzyme 3A4; RCT, randomized controlled trial)

Figure S9. Effect on LDL-C between CYP3A4 statin + clopidogrel and non-CYP3A4 statin + clopidogrel (CYP3A4, cytochrome P450 isoenzyme 3A4; RCT, randomized controlled trial)

Figure S10. Effect on HDL-C between CYP3A4 statin + clopidogrel and non-CYP3A4 statin + clopidogrel (CYP3A4, cytochrome P450 isoenzyme 3A4; RCT, randomized controlled trial)

Figure S11. Effect on TC between CYP3A4 statin + clopidogrel and non-CYP3A4 statin + clopidogrel (CYP3A4, cytochrome P450 isoenzyme 3A4; RCT, randomized controlled trial)

Figure S12. Effect on death between CYP3A4 statin + clopidogrel and non-CYP3A4 statin + clopidogrel (CYP3A4, cytochrome P450 isoenzyme 3A4; RCT, randomized controlled trial)

Figure S13. Effect on myocardial infarction between CYP3A4 statin + clopidogrel and non-CYP3A4 statin + clopidogrel (CYP3A4, cytochrome P450 isoenzyme 3A4; RCT, randomized controlled trial)

Figure S14. Effect on stroke between CYP3A4 statin + clopidogrel and non-CYP3A4 statin + clopidogrel (CYP3A4, cytochrome P450 isoenzyme 3A4; RCT, randomized controlled trial)

Figure S15. Effect on major adverse cardiovascular events between CYP3A4 statin + clopidogrel (CYP3A4, cytochrome P450 isoenzyme 3A4; RCT, randomized controlled trial)


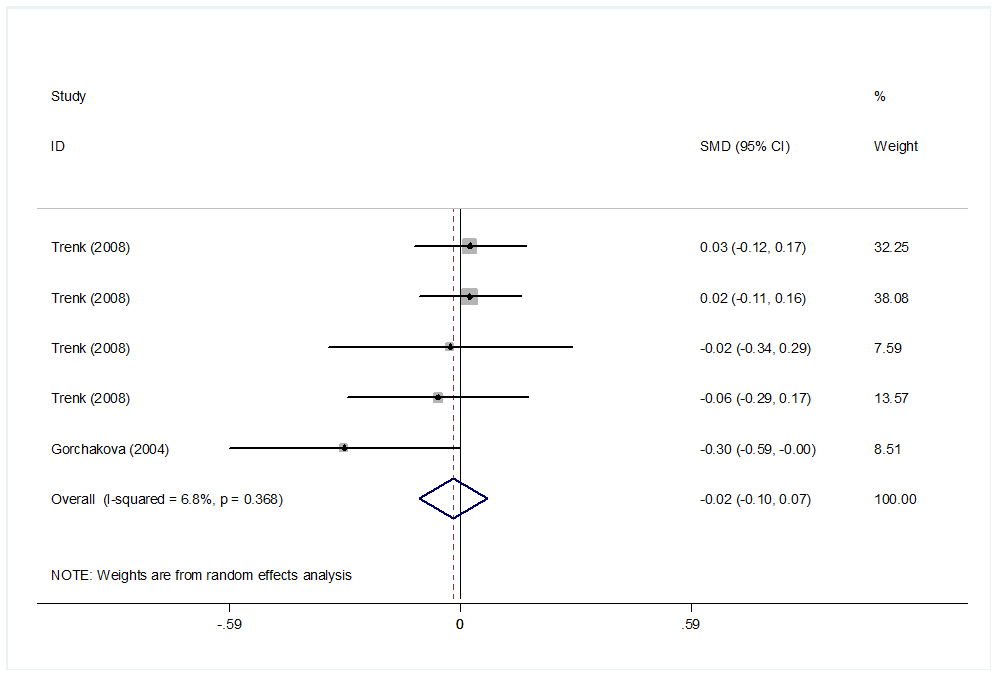


Figure S1. Effect on residual platelet aggregation indicator between statin + clopidogrel and clopidogrel.


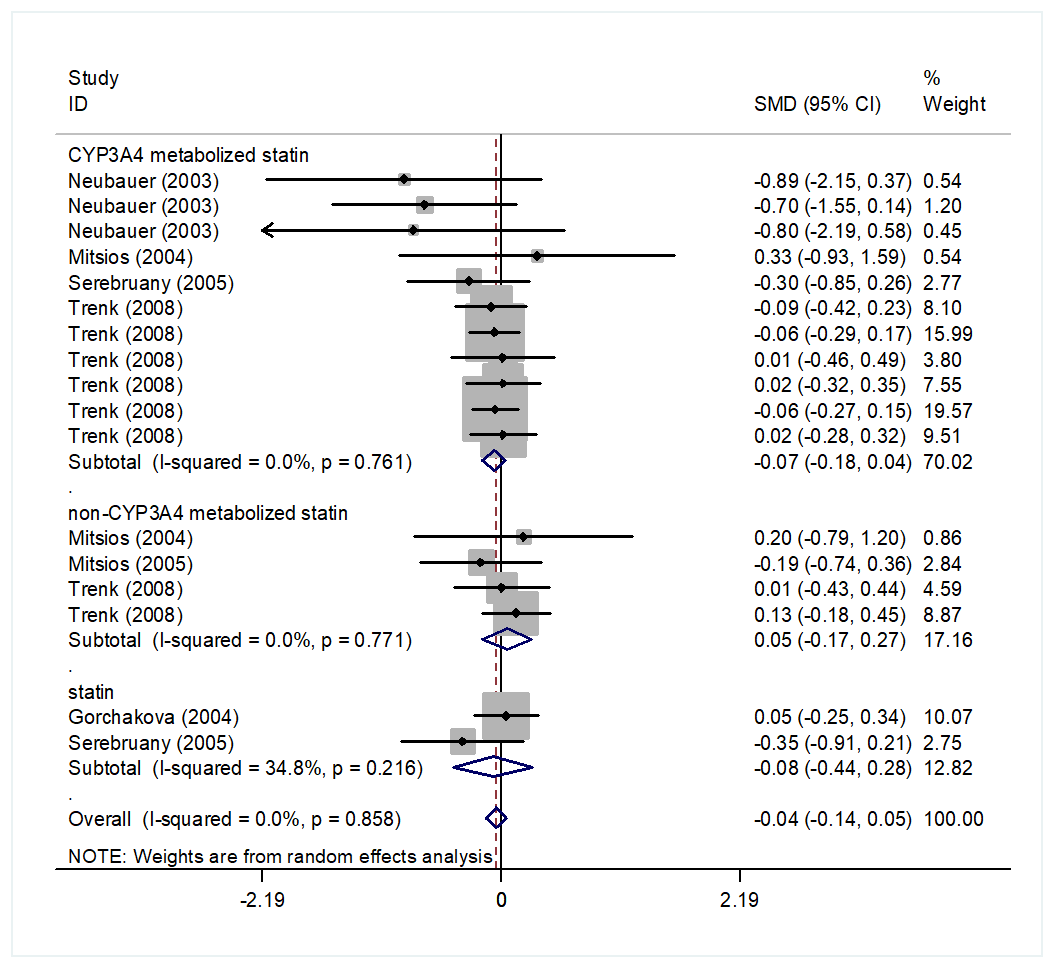


Figure S2. Effect on P-selectin between statin + clopidogrel and clopidogrel (CYP3A4, cytochrome P450 isoenzyme 3A4)


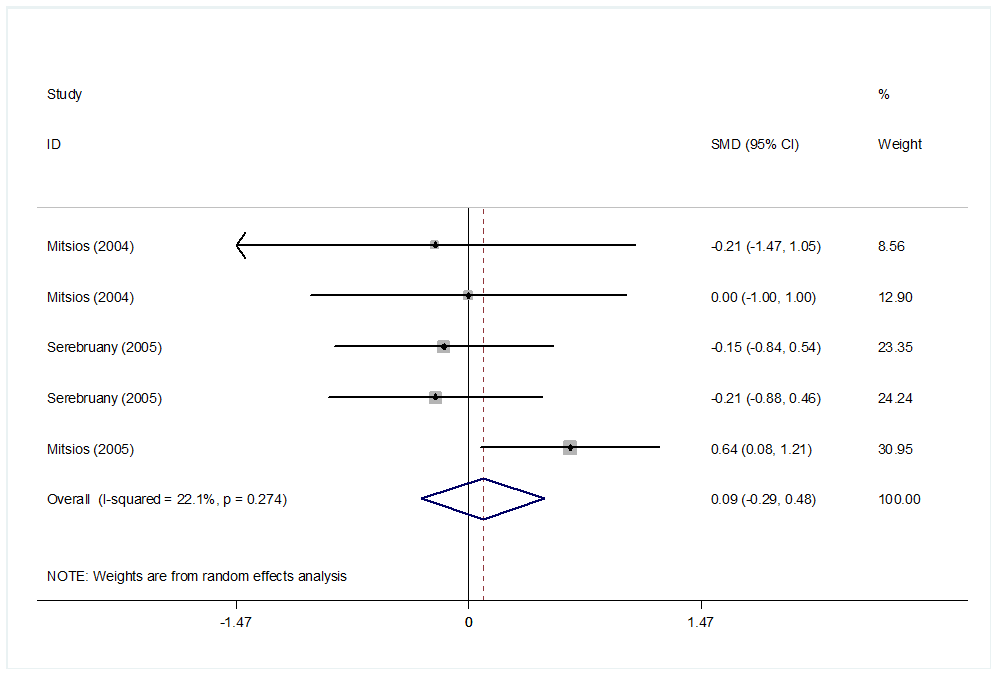


Figure S3. Effect on CD40L between statin + clopidogrel and clopidogrel


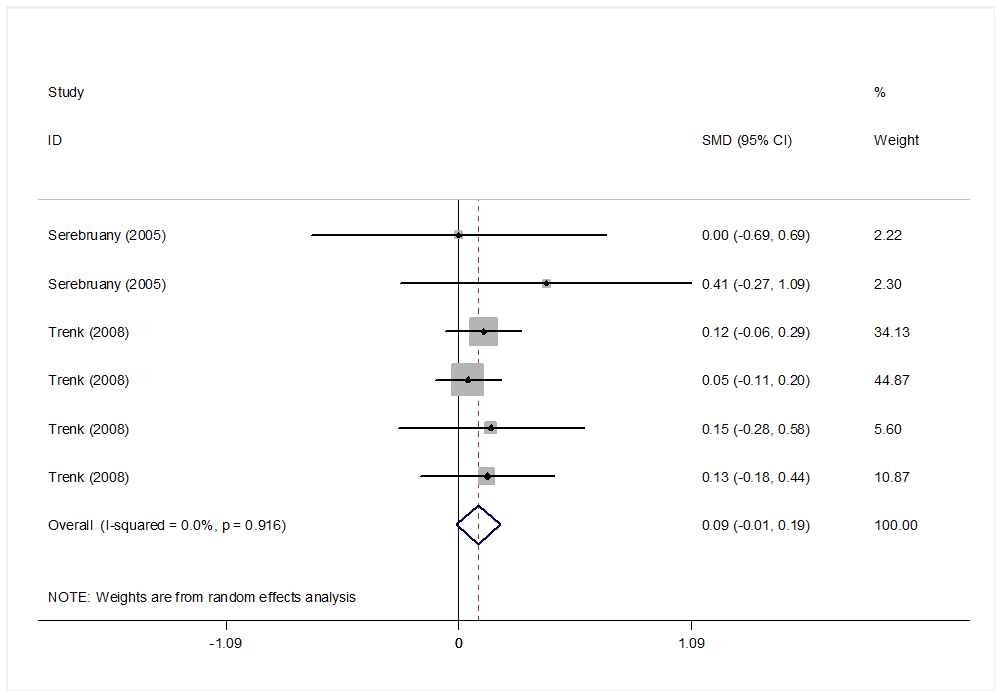


Figure S4. Effect on CD63 (LAMP-3) between statin + clopidogrel and clopidogrel


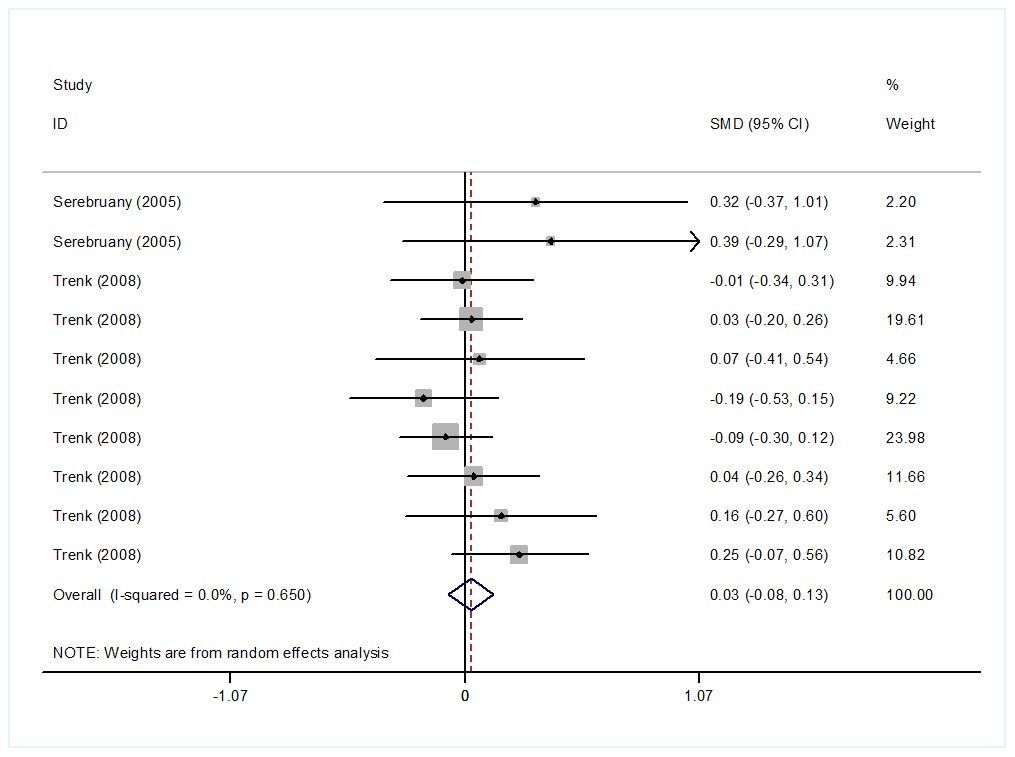


Figure S5. Effect on PAC-1 between statin + clopidogrel and clopidogrel


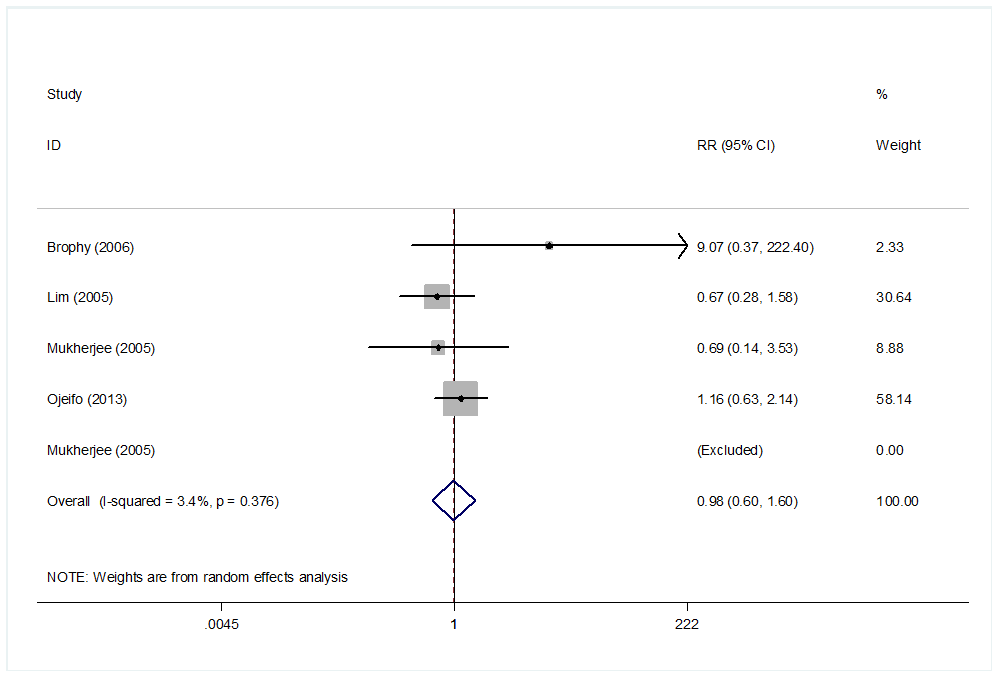


Figure S6. Effect on myocardial infarction between statin + clopidogrel and clopidogrel


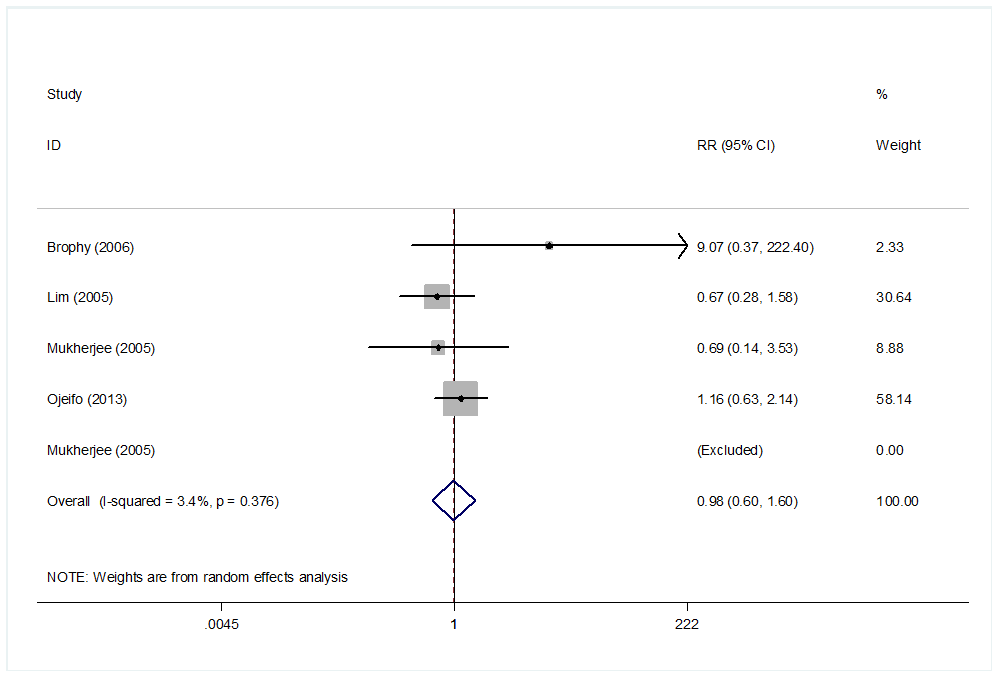


Figure S7. Effect on stroke between statin + clopidogrel and clopidogrel


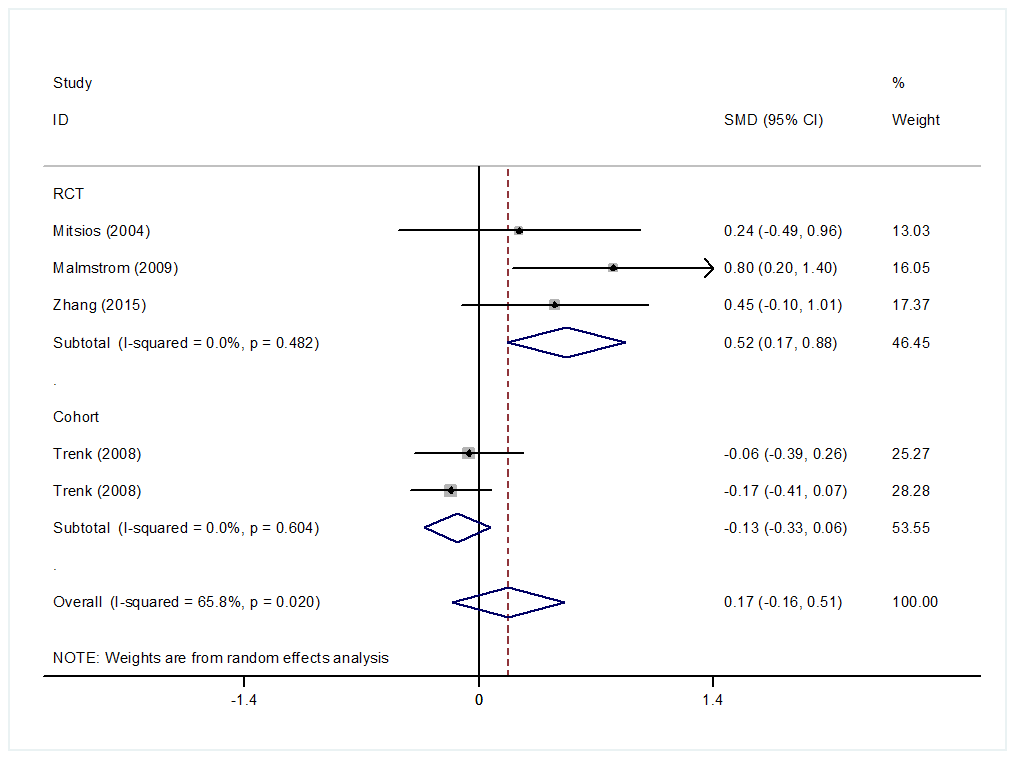


Figure S8. Effect on P-selectin between CYP3A4 statin + clopidogrel and non-CYP3A4 statin + clopidogrel (CYP3A4, cytochrome P450 isoenzyme 3A4; RCT, randomized controlled trial)


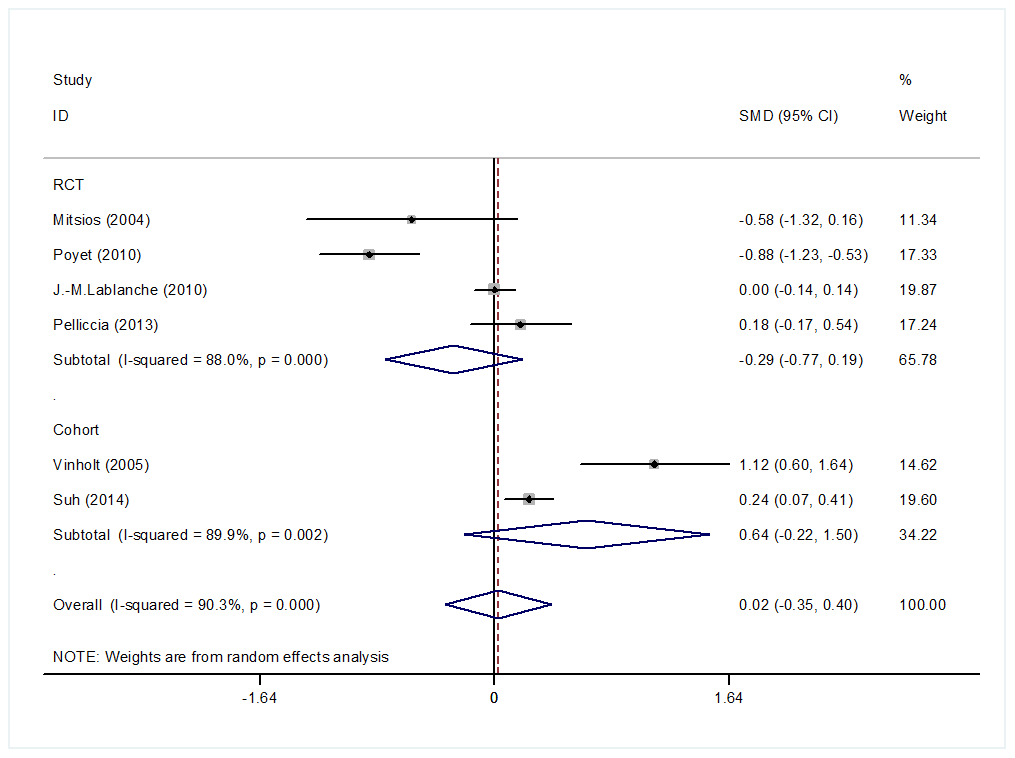


Figure S9. Effect on LDL-C between CYP3A4 statin + clopidogrel and non-CYP3A4 statin + clopidogrel (CYP3A4, cytochrome P450 isoenzyme 3A4; RCT, randomized controlled trial)


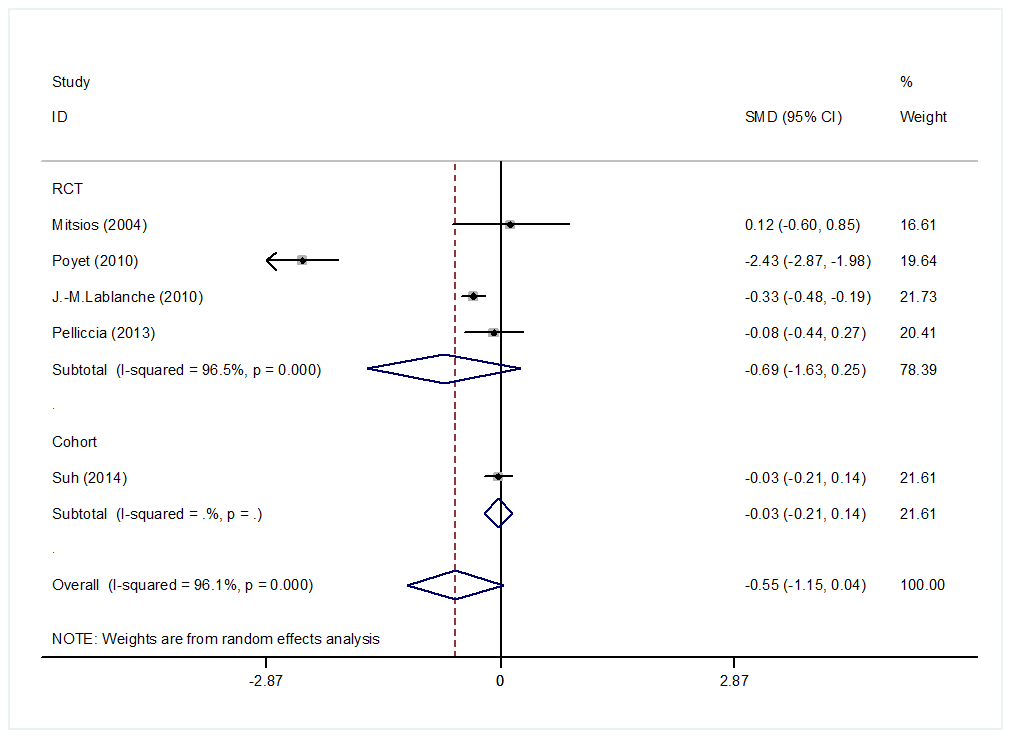


Figure S10. Effect on HDL-C between CYP3A4 statin + clopidogrel and non-CYP3A4 statin + clopidogrel (CYP3A4, cytochrome P450 isoenzyme 3A4; RCT, randomized controlled trial)


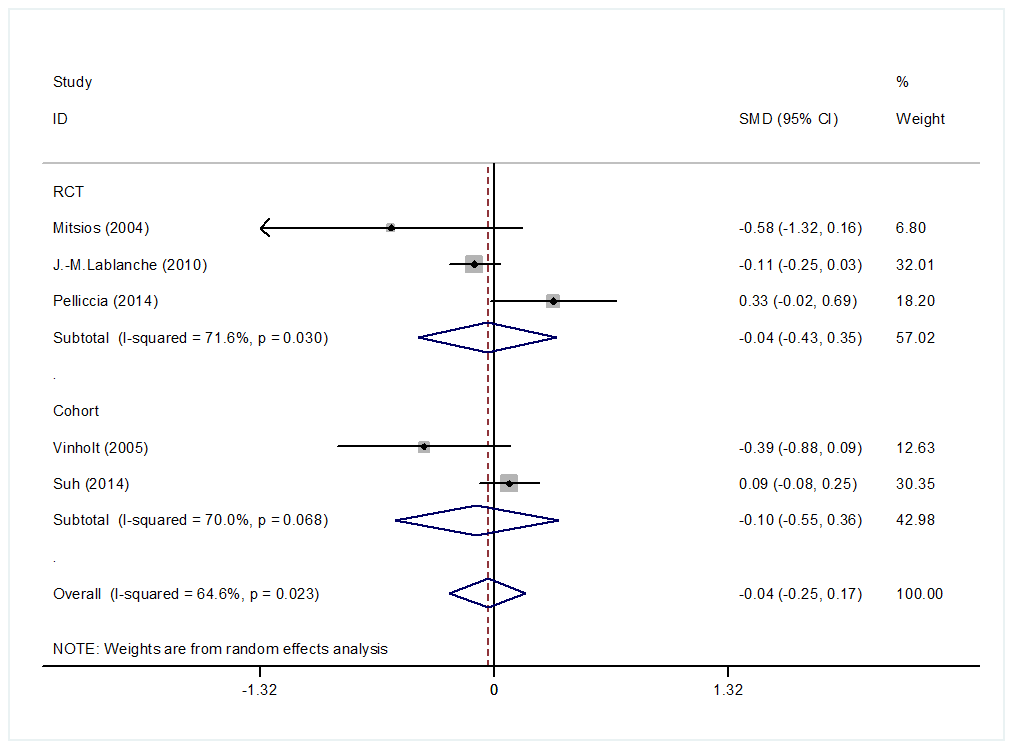


Figure S11. Effect on TC between CYP3A4 statin + clopidogrel and non-CYP3A4 statin + clopidogrel (CYP3A4, cytochrome P450 isoenzyme 3A4; RCT, randomized controlled trial)


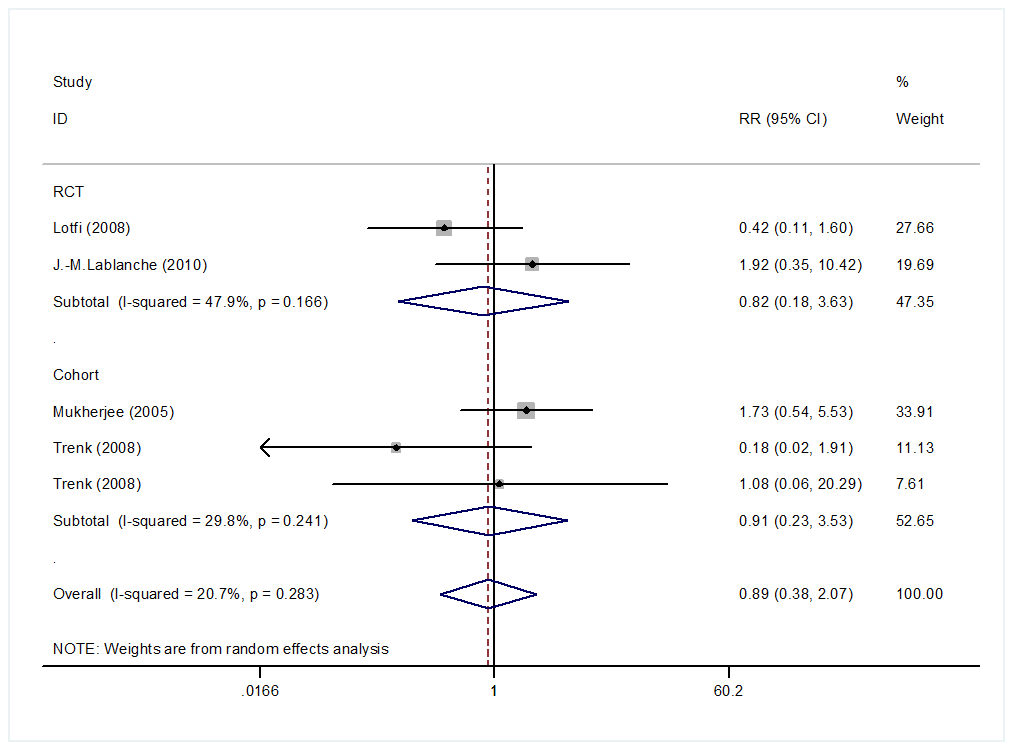


Figure S12. Effect on death between CYP3A4 statin + clopidogrel and non-CYP3A4 statin + clopidogrel (CYP3A4, cytochrome P450 isoenzyme 3A4; RCT, randomized controlled trial)


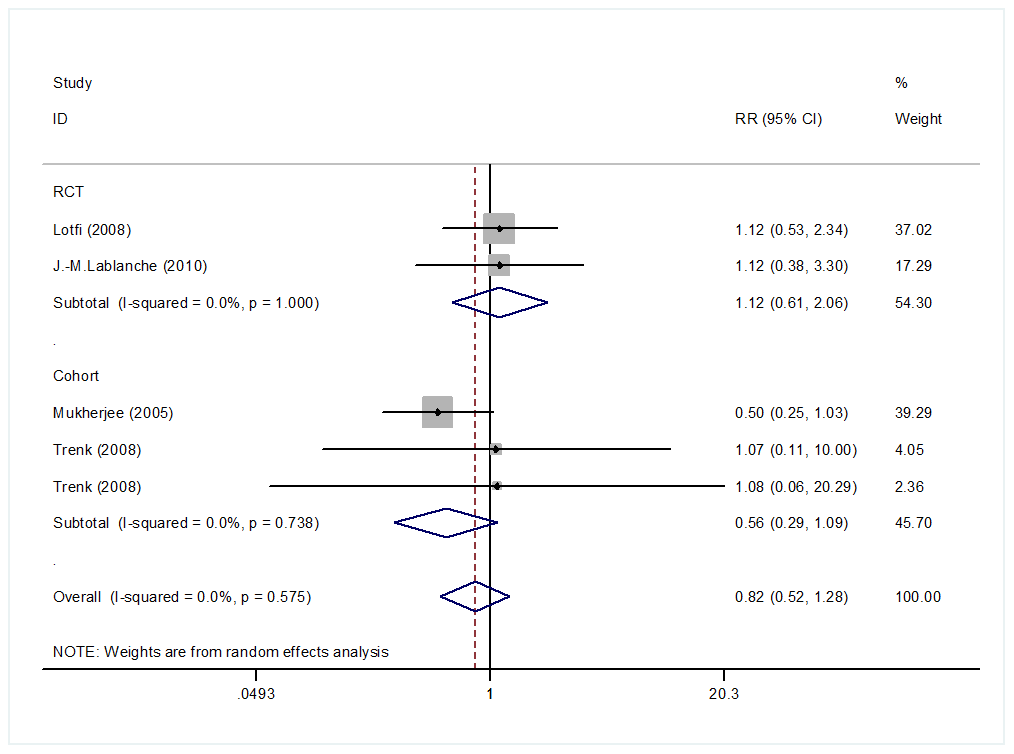


Figure S13. Effect on myocardial infarction between CYP3A4 statin + clopidogrel and non-CYP3A4 statin + clopidogrel (CYP3A4, cytochrome P450 isoenzyme 3A4; RCT, randomized controlled trial)


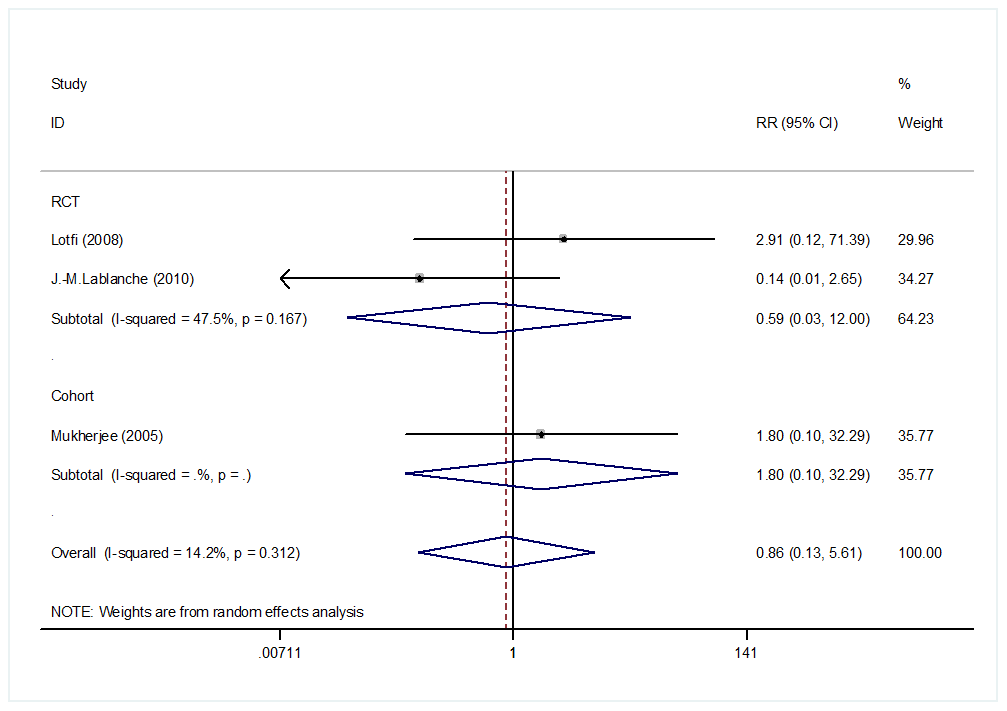


Figure S14. Effect on stroke between CYP3A4 statin + clopidogrel and non-CYP3A4 statin + clopidogrel (CYP3A4, cytochrome P450 isoenzyme 3A4; RCT, randomized controlled trial)


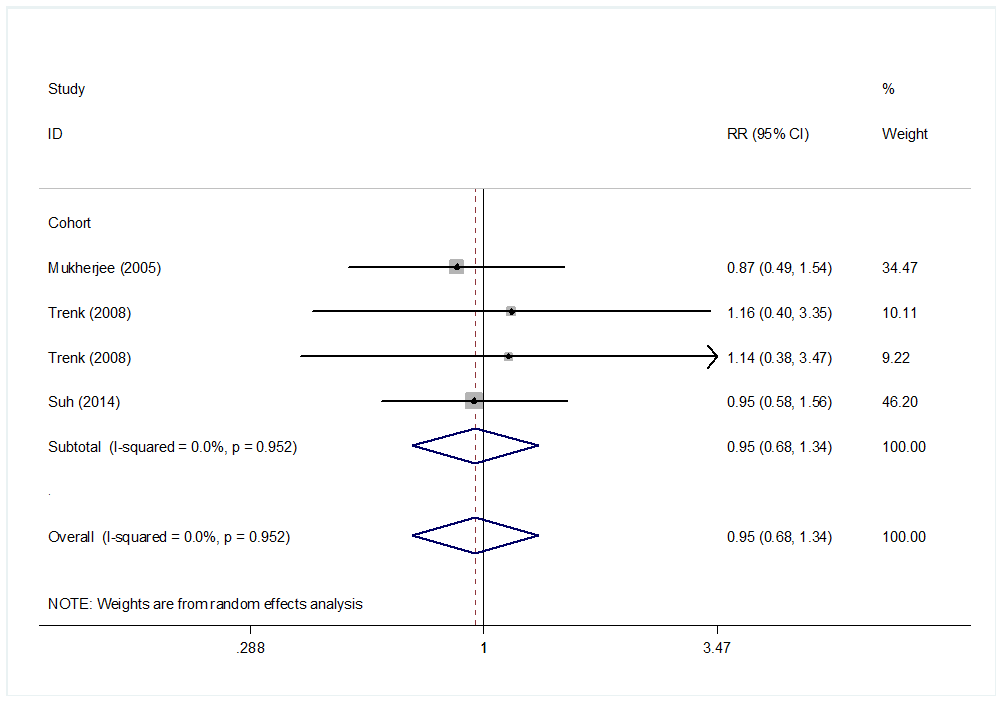


Figure S15. Effect on major adverse cardiovascular events between CYP3A4 statin + clopidogrel (CYP3A4, cytochrome P450 isoenzyme 3A4)
